# Advanced Macromolecular Architectures via Inorganic Polymers

**DOI:** 10.1002/marc.202500615

**Published:** 2025-11-21

**Authors:** Edip Ajvazi, Pauline Stadler, Paul Strasser, Ian Teasdale

**Affiliations:** ^1^ Institute of Polymer Chemistry Johannes Kepler University Linz Austria

**Keywords:** inorganic polymers, macromolecular architectures, polyphosphazenes, polyphosphoesters, polysiloxanes

## Abstract

Advanced macromolecular architectures, extending beyond mere chemical composition, are key to unlocking new functionalities in inorganic polymers. This review highlights recent advances in the design and synthesis of inorganic polymers with complex architectures, ranging from hyperbranched and graft polymers to dendrimers, such as hyperbranched polyphosphoester, star‐branched polyphosphazenes and polydimethylsiloxane bottlebrushes, thus extending well beyond traditional linear chains. These structural motifs enable unique and tunable properties, such as degradation profiles and mechanical performance, expanding the range of applications in biomedical and technical fields. Particular emphasis is placed on synthetic strategies that enable precise architectural control. While such structural diversity is well established in organic systems, this review focuses on inorganic polymers featuring main‐group elements in the polymer backbone and as key structural elements. Phosphorus‐ and silicon‐based polymers, especially polyphosphazenes, polyphosphoesters, and polysiloxanes, constitute the majority of studied systems and are covered in depth, alongside emerging classes incorporating sulfur, tin, selenium, and metallocenes.

## Graphical abstract

From linear chains to advanced macromolecular architectures, this review explores how innovations in inorganic polymers unlock new structural and functional capabilities. Emphasizing modern synthetic strategies and unique architectural designs, it highlights the emerging potential of main‐group element systems as next‐generation alternatives to traditional carbon‐based materials.

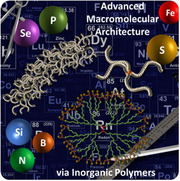

## 1 Introduction

Polymer chemistry has evolved from simple, undefined linear and branched structures toward precisely designed macromolecular entities. Indeed, today's polymers are not solely defined by their chemical identity but more and more by the spatial connectivity of their monomeric units, their precise sequential configuration, and, thereby, their overall tertiary and quaternary structure. Over the last few decades, the development and ongoing improvement of controlled and living polymerization methods significantly strengthened the field of complex, higher macromolecular architecture and engineering [1, 2, 3, 4, 5]. Nevertheless, the abundance of reported polymerization techniques and macromolecular systems is designed for organic polymers. In contrast, literature regarding higher architectural design of inorganic polymers is limited [6, 7].

Inorganic polymers, either solely based on inorganic elements in their backbone or in combination with carbon, provide a unique set of properties, not attainable with organic polymers [6]. Given the broad selection of main group elements, a wide variety of materials can be synthesized. Silicon and phosphorus are predominantly utilized as the key structural components in these polymers, with polydimethylsiloxane (PDMS) being the most prominent commercial representative [8]. Although research regarding the macromolecular engineering and architectural design of these materials is fairly recent compared to carbon‐based systems, the implications of such materials in various fields of application are extensive. While not exhaustive, this review aims to showcase selected examples of inorganic polymers and their utilization in advanced macromolecular architectures, as well as the implementation of inorganic elements as integral parts of the architectural design. In contrast to previous reviews, this review uniquely examines inorganic elements and their distinctive contributions to macromolecular engineering

## 2 Macromolecular Architecture and Engineering

A broad variety of different macromolecular architectures has become available via the development of controlled and precise polymerization methods, ranging from fully defined dendrimers, over graft polymers and branched architectures to less‐defined but highly branched hyperbranched polymers. Arguably, the simplest architecture in this category would be linear block co‐polymers, either with only two segments or more. However, mostly applicable in the field of supramolecular self‐assembly, linear block co‐polymers will not be discussed in the context of this review. Instead, focusing in detail on higher macromolecular architectures, readers may be referred to other excellent reviews for detailed insights into linear systems [9, 10, 11]. In general, each of the discussed architectures presents distinct advantages compared to one another, while requiring specific synthetic approaches and facing individual trade‐offs relating to design complexity and accessibility. The following section will briefly describe the architectures presented herein.

**FIGURE 1:**
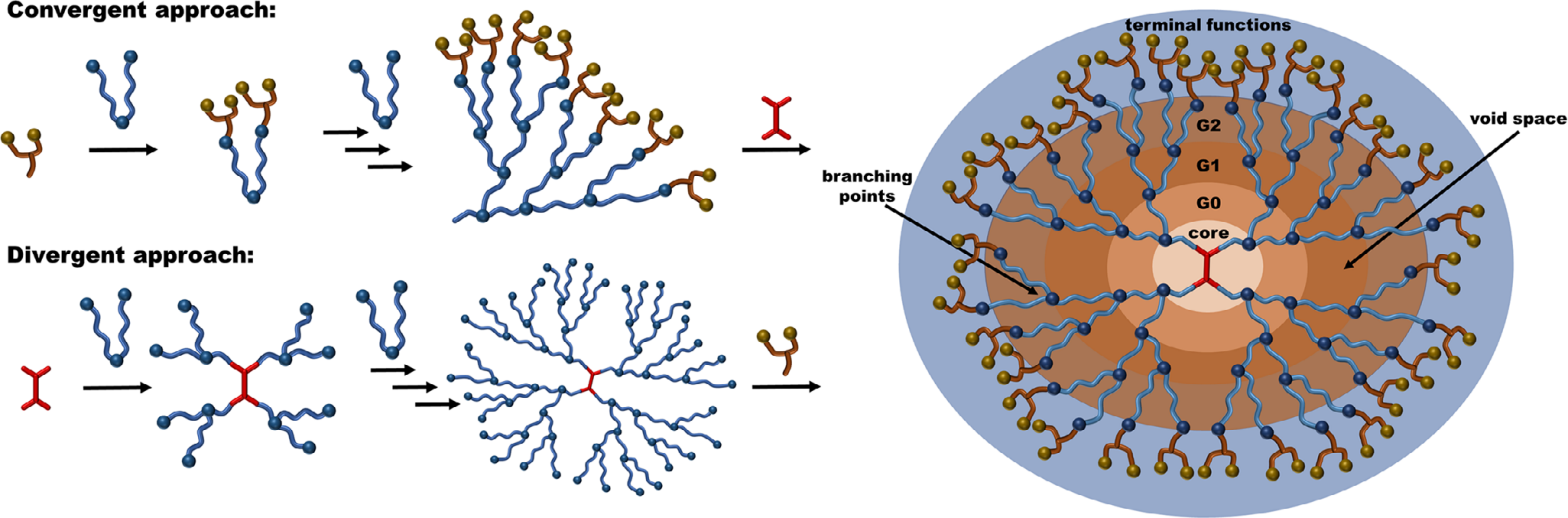
Schematic representation of convergent and divergent synthetic approaches for dendrimer formation, including the general structure and key architectural features.

Dendrimers represent the pinnacle of architecturally defined synthetic materials. They are typically synthesized in a stepwise fashion, either via a divergent approach, starting from a multivalent core and iteratively synthesizing each layer, or a convergent approach, constructing the dendrons from the outer layer inward, followed by the attachment to a core, Figure 1 [12, 13]. Both strategies necessitate complete conversion of the branching units at every layer, demanding extensive purification methods to ensure the symmetric growth of the dendritic structure. Additionally, orthogonal chemistries are essential to prevent unwanted side reactions or uncontrolled polymerization at the branching sites. Consequently, the synthesis of dendrimers is often tedious and time‐consuming, increasing exponentially in complexity with each generation. Despite this, dendrimers provide unmatched control over the architecture, resulting in monodisperse structures with highly tunable surface functionalization, rendering them interesting materials, for example, in the biomedical field [14, 15].

**FIGURE 2:**
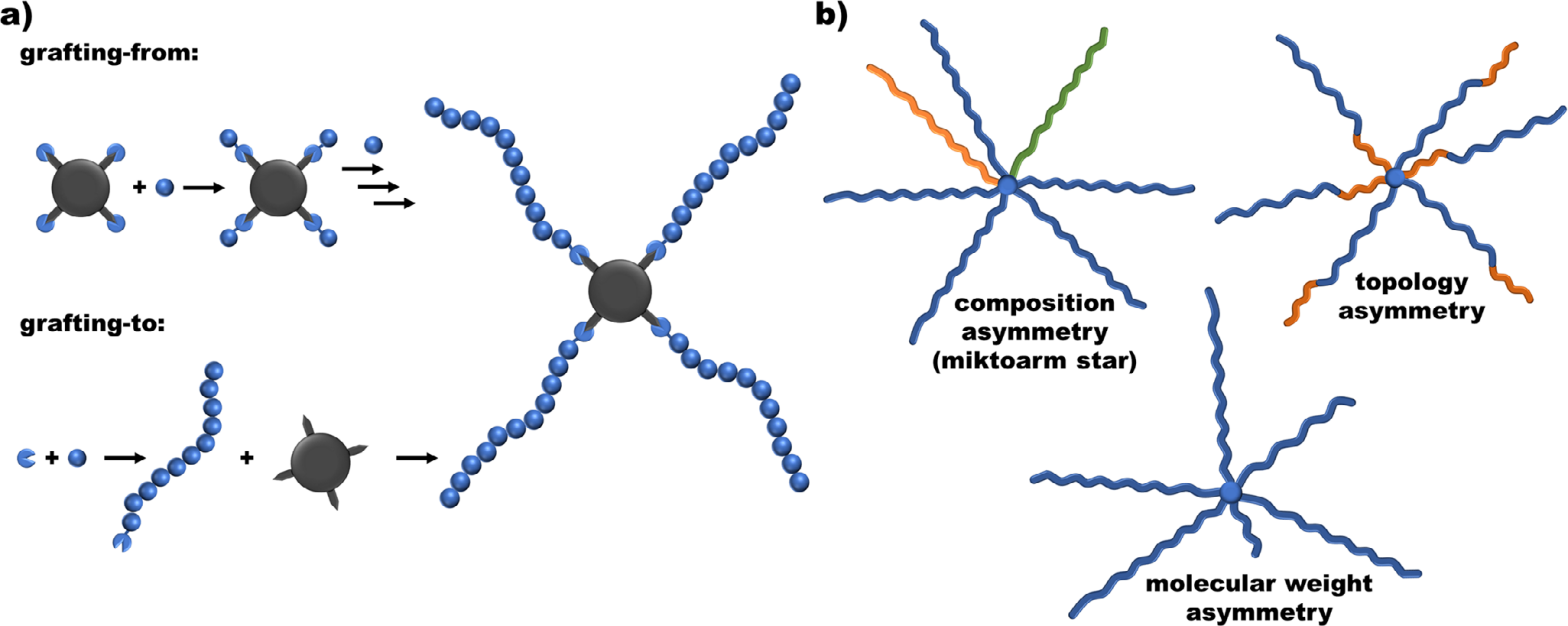
(a) Synthetic approaches for the formation of star‐branched polymers. (b) Exemplary architectural designs of asymmetric star‐branched (co‐)polymers.

Analogous to dendrimers, star‐branched macromolecular architectures grow from a distinct core that defines their spatial design and ultimately determines the number of functional groups in the final construct. They can be built up either through a “grafting‐from,” where the core acts as the initiator for polymer chain growth, or a “grafting‐to” approach, wherein pre‐formed and characterized side chains are reacted onto the core, Figure 2a [16, 17, 18]. Star‐branched polymers further offer greater versatility and tunability, allowing for the design of symmetric structures with identical (co‐)polymer arms, as well as asymmetric stars that vary in chemical composition, molecular weight, or topology between their arms, Figure 2b [1, 18]. Compared to dendrimers, which require precise stepwise synthesis, star‐branched polymers allow for broader tunability, such as increased molecular weights or chemical diversity, while maintaining a simpler and more direct synthetic process at the cost of less structural precision.

**FIGURE 3:**
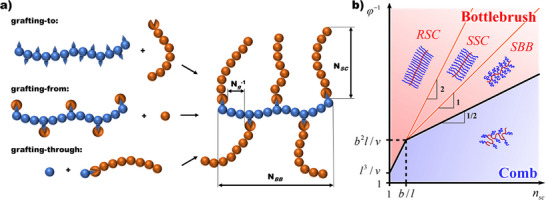
(a) Synthetic pathways toward bottlebrush polymers and their key architectural parameters. N*_BB_*—Degree of polymerization of the backbone polymer; N*_SC_*—degree of polymerization of the side chain polymer; N*_g_* ^−1^ ‐ grafting density. (b) Classification of graft‐polymers in the melt based on their architectural parameters in logarithmic scales. RSC—rod‐like side chain regime; SSC—stretched side chain regime; SBB—stretched backbone regime; b—Kuhn length; l—bond length; v—excluded volume of the monomer; φ—molar fraction of the backbone monomers. Reprinted with permission from [21]. Copyright 2017 American Chemical Society.

Graft polymers consist of a linear backbone with polymeric side chains extending from it. Similar to star‐branched materials, graft‐polymers can be synthesized through three main approaches: “grafting‐to,” where end‐functionalized polymers are reacted onto a macromolecular backbone bearing reactive pendant groups; “grafting‐from,” where the side chains are directly polymerized from a backbone decorated with initiating species; or “grafting‐through,” which involves polymerization of end‐functionalized side chains bearing polymerizable groups and acting as macromonomers to form the backbone in the process, Figure 3a [19]. As with the star‐branched architecture, graft polymers offer extensive tuneability and are defined not only by their chemical composition but also by their structural characteristics, namely the degree of polymerization of both backbone (N*_BB_*) and side chain (N*_SC_*) polymers, as well as the number of backbone bonds between side chains, or grafting density (N*_g_* or N*_g_* ^−1^, respectively), Figure 3a. These parameters in combination determine the overall spatial arrangement and result in comb‐like polymers or macromolecular bottlebrushes, the latter exhibiting a stretched‐out backbone in contrast to an unperturbed chain, Figure 3b [20, 21, 22]. Particularly in recent years, graft polymers have gained interest in various research fields, for example, employing bottlebrush polymers as nanocarriers for drug delivery or as components in elastomer formulations for energy dissipation [22, 23, 24, 25].

**FIGURE 4:**
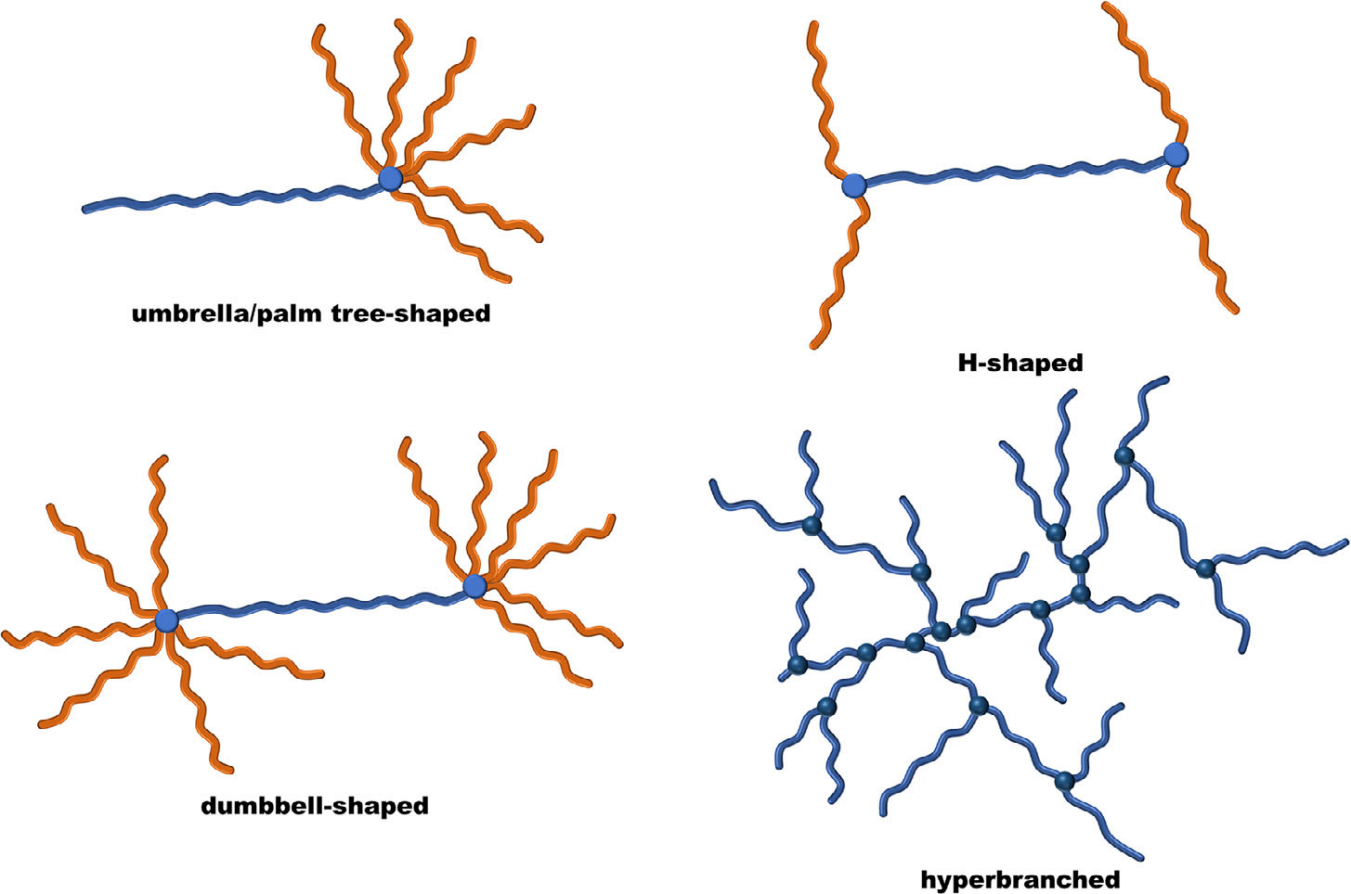
Examples of branched and hyperbranched macromolecular structures.

Finally, branched and hyperbranched structures cover a plethora of specially designed architectures, requiring extensive control over the polymerization and end‐group functionality [1, 2]. While mostly based on organic polymers, inorganic functionalities may act as an integral part of the architectural design, providing essential multifunctionality and accessible chemistry [6, 7]. For instance, ω‐branched or α,ω‐branched structures are accessible through tight control of the chain ends, enabling the formation of architectures such as umbrella‐shaped [26, 27], H‐shaped [28, 29], or dumbbell (co‐)polymers [30, 31], Figure 4. Hyperbranched structures sit at the boundary between defined and undefined macromolecular architectures. Although their synthesis can hardly be controlled and their precise structural characterization is challenging, their compelling properties, such as a globular shape, excellent solubility, and multifunctionality, have led to their application in various fields, including adhesives, modifiers, or flame retardants, as well as in biomedicine [32, 33, 34, 35].

## 3 Phosphorus‐Based Architectures

**FIGURE 5:**
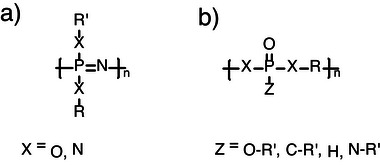
General structures of (a) PPz and (b) phosphate‐based polymers such as PPE or polyphosphoamidates (PPA).

Phosphorus‐based polymers have gained increasing attention in recent years due to their diverse properties and applications. The most studied and utilized phosphorus‐containing polymers include polyphosphazenes (PPz) and phosphate‐based polymers such as polyphosphoesters (PPE), whose respective general structures are depicted in Figure 5. This growing interest in phosphorus‐containing polymers is partly inspired by their crucial role in nature, attributed to their potential reversible binding, utilized in energy storage and metabolic control, or multivalency, exploited in the backbone construction of DNA or RNA, and which makes them an ideal building block for branched macromolecular architectures [36, 37, 38].

### 3.1 Polyphosphazenes

The most common synthesis route toward PPz is via the macromolecular precursor poly(dichloro)phosphazene. The final poly(organo)phosphazenes are obtained after a macromolecular substitution reaction, substituting the labile Cl‐moieties with suitable amine‐ or alcohol‐bearing organic substituents. Traditionally, a ring‐opening polymerization (ROP) of the cyclic trimer hexachlorophosphazene ([NPCl_2_]_3_) is employed for the formation of the macromolecular precursor. However, this reaction proceeds at elevated temperatures of around 250°C for several hours under vacuum, limiting the control over molecular weight and dispersity (Đ). Synthetic advancements, namely the development of the living cationic polymerization, have opened pathways to controlled molecular weights, narrow dispersities, and more complex molecular architectures [39, 40].

#### 3.1.1 Graft Polymers

**FIGURE 6:**
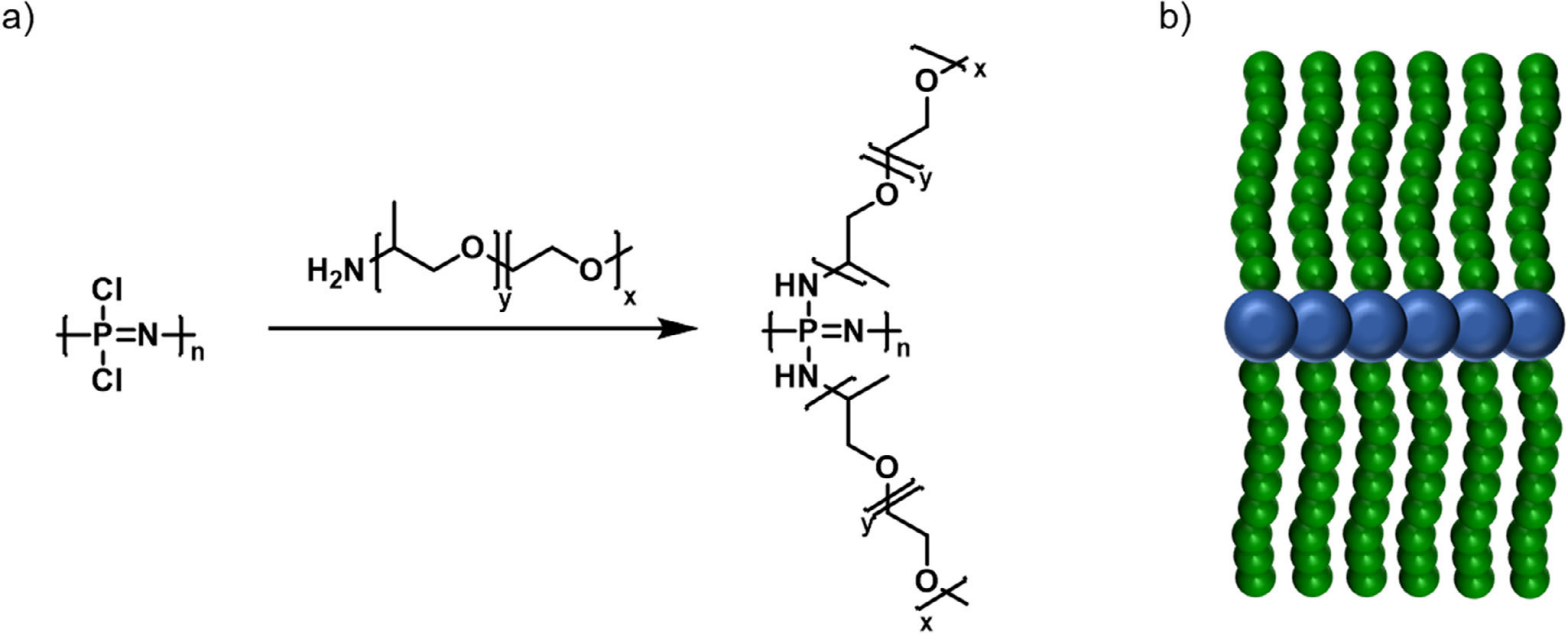
PPz‐brush formation via a grafting‐to approach described in literature [41]; (a) Substitution reaction of the precursor poly(dichlorophosphazene) with a mono amine‐functionalized poly(ethylene oxide)/poly(propylene oxide)copolymer. (b) Schematic illustration of the formed bottlebrush.

Based on the aforementioned macromolecular substitution reaction, comb‐like and bottlebrush polymers in particular are readily available via a grafting‐to approach. For example, a commercial mono amine‐functionalized Jeffamine, a poly(ethylene oxide)/poly(propylene oxide) random copolymer, can be directly grafted onto a PPz‐backbone (Figure 6) [41]. The obtained polymers exhibit thermoresponsive behavior, enabling triggered self‐assembly, and are of particular interest for biomedical applications such as drug delivery or as micromotors [42, 43]. Given the availability of two labile chlorine atoms per repeating unit for substitution, a remarkably high grafting density of two can be achieved for PPz when compared to organic polymers, as highlighted in more detail below.

**FIGURE 7:**
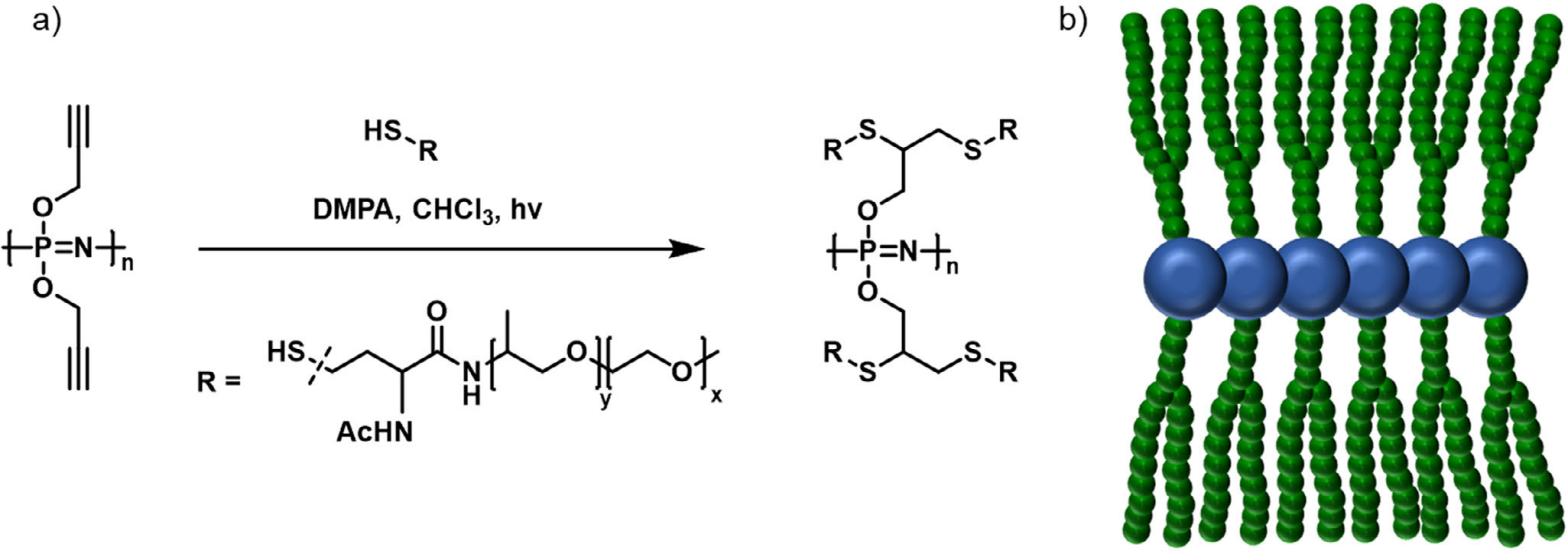
(a) PPz bottlebrush‐formation via thiol‐yne addition in a grafting‐to approach as described in literature [45], enabling exceptional grafting densities of four side‐chains per backbone repeating unit. (b) Graphical illustration of the formed PPz‐brush.

In another example, PPz with pendant unsaturated carbon moieties were prepared, using allylamine or propargyl alcohol as substituents [44, 45, 46]. Not only can one incorporate various side chains to tune functionality and properties of the final graft‐polymer via simple thiol‐ene/yne addition, including the incorporation of 3‐mercapto‐1,2‐propanediol, 2‐mercaptoethoxy ethanol, or l‐cysteine [44], but also the architectural versatility can be enhanced by increasing the grafting density even more, up to 4 side‐chains per repeating unit, Figure 7 [45].

**FIGURE 8:**
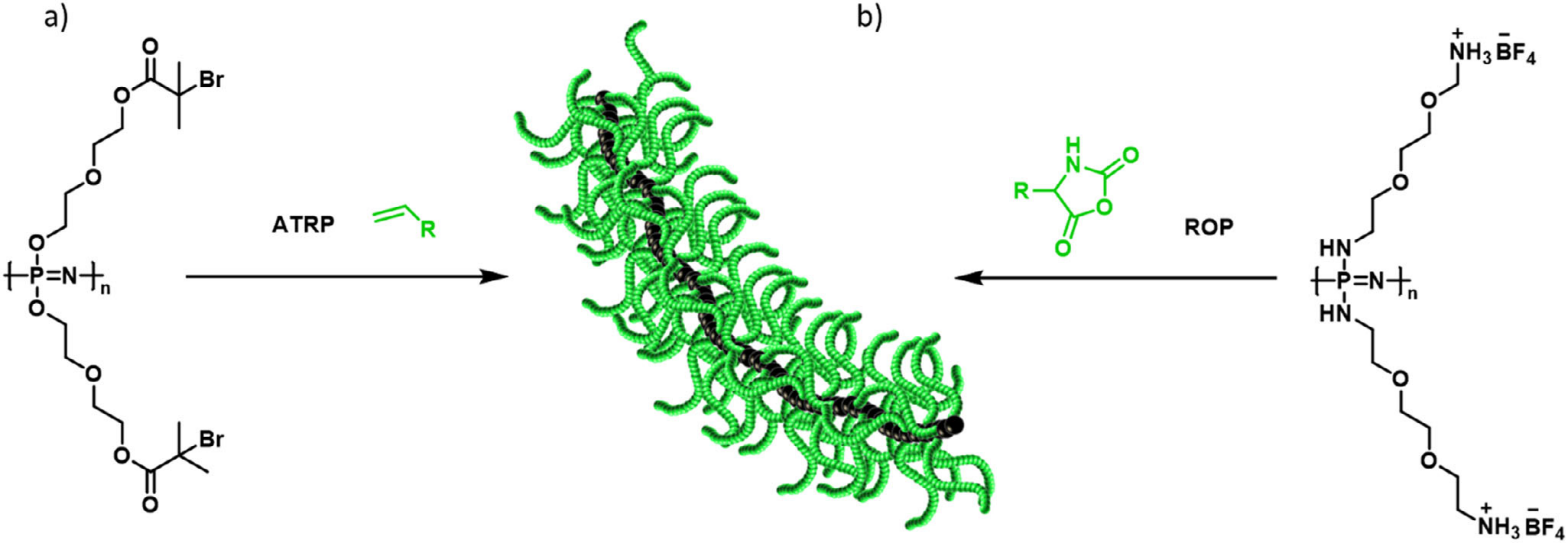
PPz macroinitiators for bottlebrush formation via a grafting‐from approach using ATRP (a) [47] and ROP (b) [23].

With the development of controlled radical polymerizations, the preparation of distinct macromolecular architectures with controlled dimensions and narrow molecular weight distributions became possible. For instance, based on atom‐transfer radical polymerization (ATRP), Allcock et al. reported various PPz graft‐co‐polymers. A PPz‐backbone bearing pendant hydroxyl groups was esterified with 2‐bromopropionyl bromide to form alkyl bromide moieties. This ATRP‐macroinitiator was subsequently used for the polymerization of polystyrene, poly(tert‐butyl acrylate), or poly(N‐isopropylacrylamide) side‐chains, and the resulting organic–inorganic graft copolymers were investigated regarding their use as thermoplastic elastomeric materials or application in the biomedical field (Figure 8a) [47].

Recently, a PPz‐*g*‐poly(α‐glutamate) bottlebrush polymer was reported as well. The PPz backbone was functionalized with a primary amine and further modified toward BF_4_‐ammonium salts, acting as the initiating moiety for controlled ROP of N‐carboxy anhydrides of glutamic acid (Figure 8b). The resulting PPz‐*g*‐poly(glutamic acid) bottlebrush polymers allowed for significantly higher molecular weights, compared to their linear poly(glutamic acid) analogues, reaching hydrodynamic diameters up to 50 nm, which is promising for applications as drug delivery agents. An increased intracellular uptake in CT26 colon cancer cells was reported, as well as enhanced pharmacokinetics by extending plasma half‐lives and reducing renal clearance [23].

**FIGURE 9:**
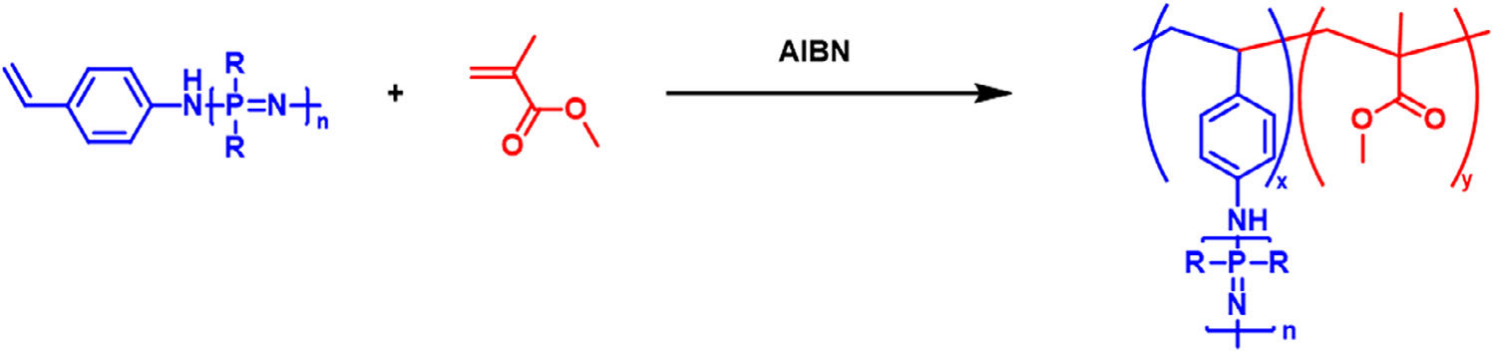
PPz‐graft co‐polymer formation via a grafting‐through approach, copolymerizing vinylaniline‐terminated poly(trifluoroethoxy)phosphazene and methyl methacrylate. For 17mol% of a PPz side chain with n = 30, M_n_ = 45100, Đ = 1.3 [49].

Grafting‐through approaches for PPz‐based graft polymers are hardly reported; one approach utilized a vinylaniline‐terminated PPz for free radical copolymerization with styrene, depicted in Figure 9 [48]. Overall, up to 15wt% of the PPz constituent was achieved; however, this only resulted in roughly 1‐2 side chains per polystyrene backbone. Grafting density could be increased in a system based on a methyl methacrylate backbone, where around 17 mol% of grafts were achieved, or even higher up to 100 mol% for norbornenyl end‐functionalized PPz [49, 50].

#### 3.1.2 Star‐Branched Polyphosphazenes

**FIGURE 10:**
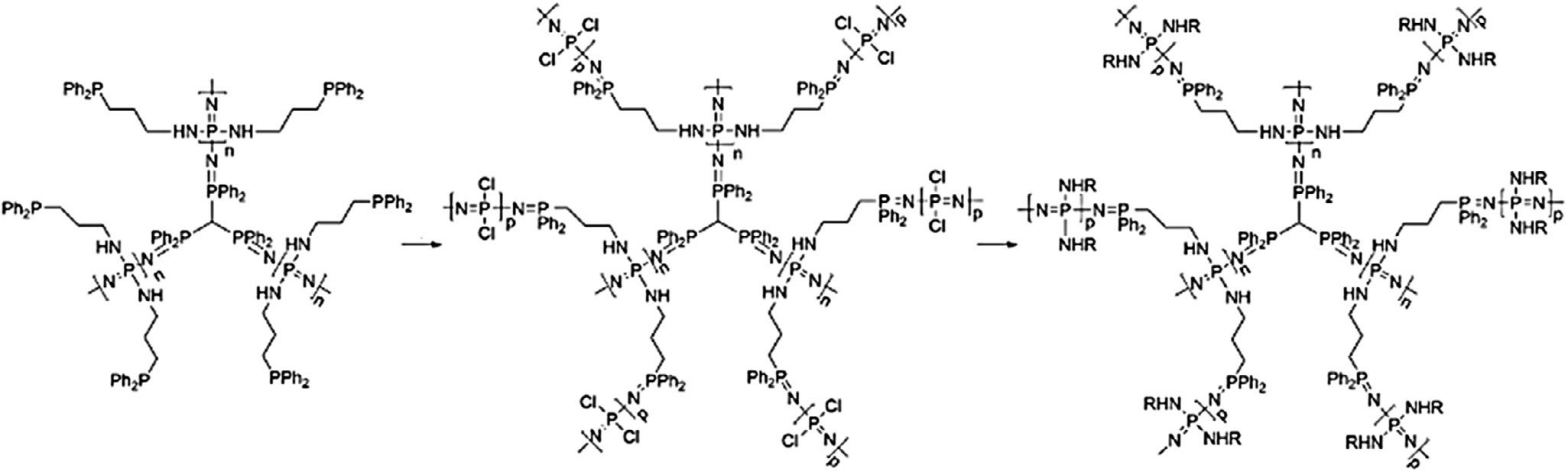
Schematic synthesis pathway toward PPz‐based star‐dendritic molecular bottlebrushes. Adapted from [51], licensed under CC BY 4.0.

Star‐branched PPz can be readily prepared via a phosphine‐mediated living cation polymerization. Using 1,1,1‐tris(diphenylphosphino)methane as the initiating core, three‐armed star polymers can be synthesized in a divergent approach and further modified during the macromolecular substitution reaction. For instance, a mono‐amine capped Jeffamine as a substituent allows for the formation of three‐arm star‐branched bottlebrush polymers [51], whereas substitution with propargyl alcohol and subsequent functionalization with 1‐thioglycerol results in the formation of dendritic polyols [52]. Extending on the reported chemistry, the formed three‐armed star‐branched poly(dichloro)phosphazene can also be substituted with 3‐(diphenylphosphino)‐1‐propylamine, Figure 10, providing initiation sites for the grafting of an additional generation of PPz side‐chains. After substitution with a mono‐amine capped Jeffamine, one obtains highly water soluble globular unimolecular structures in the region of 70 nm with an outstandingly large number of 30 000 end groups and potential use as materials for polymer therapeutics [51].

**FIGURE 11:**
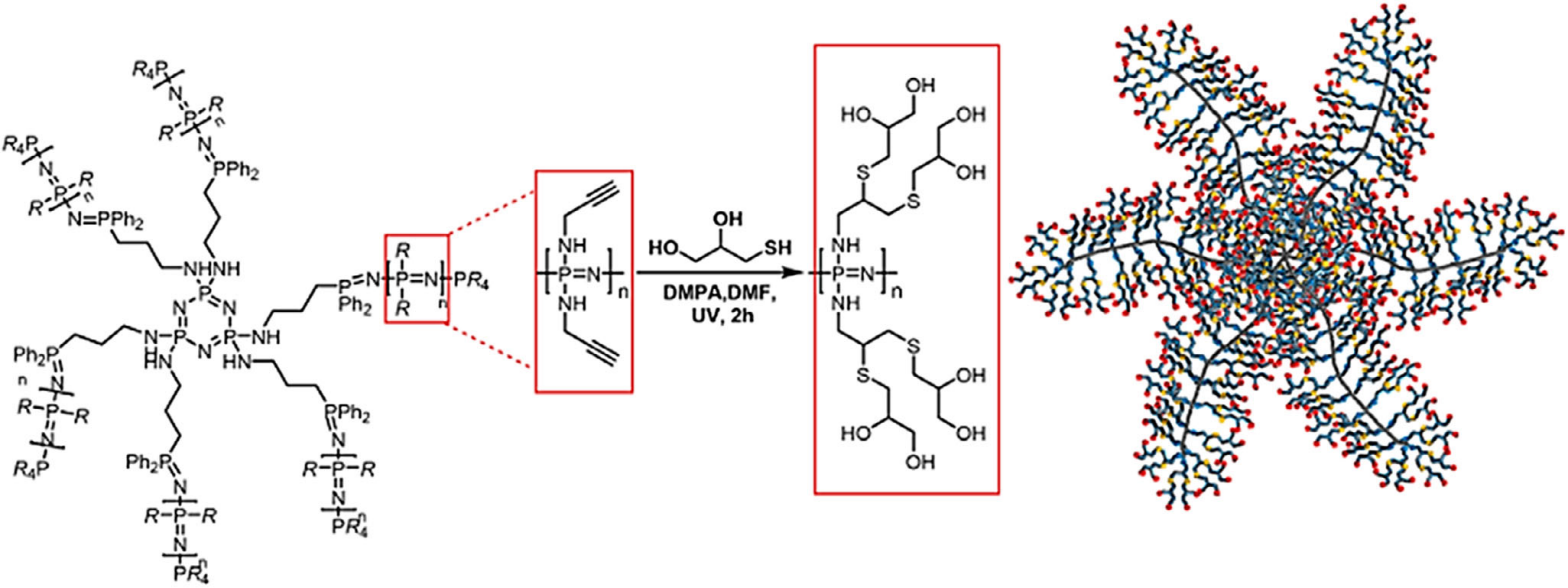
Six‐arm star‐branched PPz polyol, synthesized via a post‐polymerization functionalization approach using propargyl alcohol as macromolecular substituent and 1‐thioglycerol. Adapted from [52], licensed under CC BY 4.0. Copyright 2018 American Chemical Society.

Combining the cyclic hexachlorophosphazene trimer with 3‐(diphenylphosphino)‐1‐propylamine as substituent provides the core structure for the formation of six‐arm star‐branched PPz [52]. Again, substituting the poly(dichloro)phosphazene arms with propargyl alcohol and functionalizing with 1‐thioglycerol yielded a highly controlled, uniquely branched architecture with over 1700 functional groups per macromolecule, Figure 11.

The cyclic hexachlorophosphazene trimer is often used as a simple core structure for star‐branched polymers, offering straightforward grafting chemistry and comparably high grafting density. For example, functionalization of the core with a 2‐bromoisobutyryl derivative provides an ATRP six‐arm initiator for the polymerization of polystyrene, poly(*tert*‐butyl acrylate), or poly(N‐isopropylacrylamide) [47]. Similarly, a six‐arm star‐branched poly(2,2,6,6‐tetramethylpiperidinyloxy‐4‐yl methacrylate) could be prepared. Employing a convergent approach, poly(ethylene glycol) (PEG) star‐branched polymers based on a hexachlorophosphazene trimer‐core could be synthesized via simple DCC/DMAP coupling, enabling statistical control over the number of arms [53].

### 3.2 Polyphosphoester and Polyphosphoramidates

**FIGURE 12:**
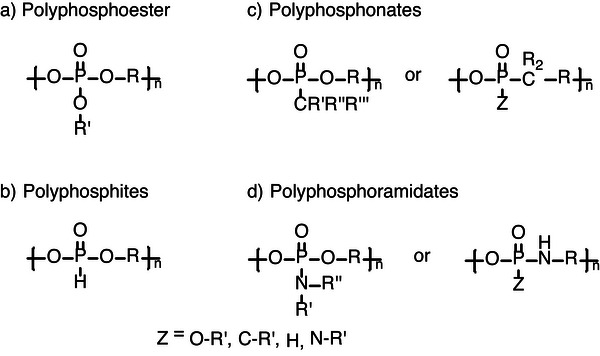
General structures of phosphate‐based polymers: (a) PPE, (b) polyphosphites, (c) polyphosphonates, and (d) PPA.

Phosphate‐based polymers are a broad class of macromolecules, generally consisting of a P═O moiety in their backbone and defined in more detail by the first binding sphere around the central pentavalent phosphorus. PPE, as the predominant sub‐class, consists of three P─O bonds on the P‐center and can be further tuned by careful selection of the residual groups in the backbone (R), as well as the side chain (R'), Figure 12a [54]. Substituting the side‐chain ester bond with hydrogen results in the formation of polyphosphites, Figure 12b. Polyphosphonates, Figure 12c, contain a P─C bond in their structure, either as part of the main chain or as the side group, providing increased stability against unwanted hydrolysis or transesterification reactions [55, 56]. In contrast, PPA includes a P─N bond in the first binding sphere of the P‐center, Figure 12c, particularly influencing the degradation characteristics of the polymer through the hydrolysis‐labile P─N bond [36, 57, 58]. Different synthetic approaches are feasible for the preparation of PPE [37], including classical polycondensation [59], polyaddition [60], ring‐opening metathesis polymerization (ROMP) [61, 62, 63], or acyclic diene metathesis (ADMET) polycondensation [63, 64], as well as ROP [63, 65].

#### 3.2.1 Hyperbranched Polymers

**FIGURE 13:**
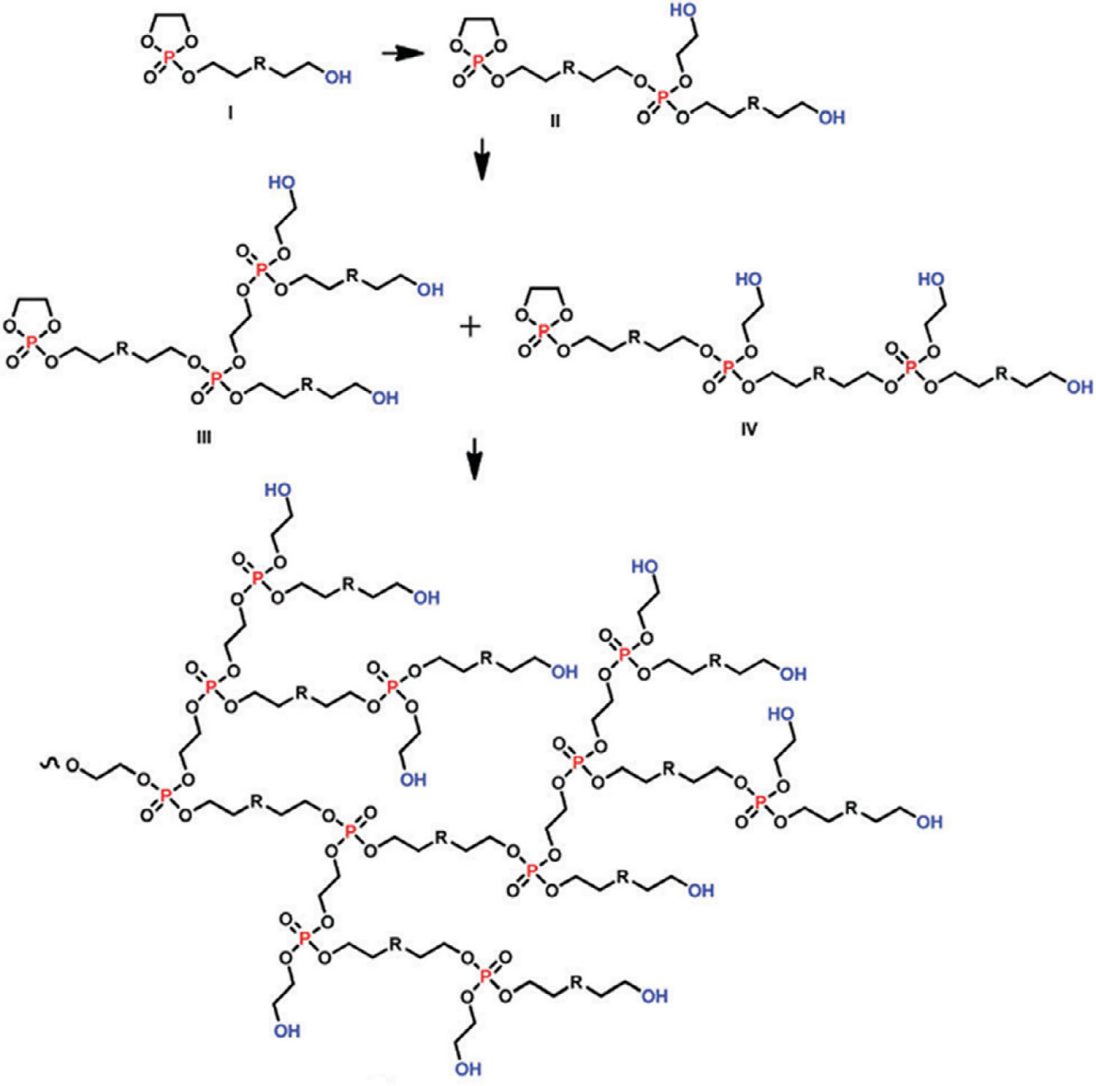
Synthesis of a hyperbranched PPE via self‐condensing ROP of a hydroxyl functionalized cyclic phosphate monomer. Adapted with permission from [65]. Copyright 2009 American Chemical Society.

Although hyperbranched polymers lack the tight control over the polymerization process compared to other branched architectures or dendrimers, they stand out due to their straightforward synthesis and the distinctive properties of these macromolecular structures. Especially for PPE, such materials are of considerable interest as biomedical materials, for example, as well as flame retardants [66]. PPEs offer various synthetic pathways toward hyperbranched structures, such as through an “A_2_+B_3_” or “AB_2_” polycondensation reaction [60, 67, 68, 69]. Subsequently, based on 2‐chloro‐2‐oxo‐1,3,2‐dioxaphospholane (COP), an AB* inimer was developed, allowing direct ring‐opening polymerization in bulk without the necessity for additional catalysts, Figure 13 [65]. Additionally, by controlling the interplay of reaction times and temperature, control over the molecular weight could be achieved. In other reports, A_3_‐type monomers based on phosphoryl chloride and vinyl alcohols, or “A_2_+CB_2_”‐systems utilizing ethyldichlorophosphate and diethanolamine have been described [70]. Given the broad topic of phosphoester hyperbranched polymers, a more detailed and in‐depth discussion can be found in additional review articles on this topic [37, 66].

#### 3.2.2 Graft Polymers

**FIGURE 14:**
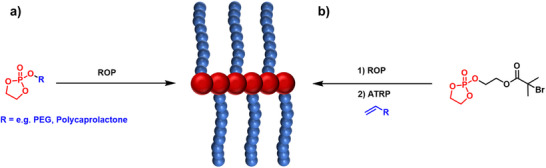
Synthetic pathways toward PPE graft‐polymers by ROP of macromonomers in a grafting‐through approach (a) [71], as well as a grafting‐through approach via pendant ATRP initiating sites (b) [72].

The preparation of various polymer bottlebrushes incorporating a PPE backbone mainly depends on the ring‐opening polymerization of cyclic phosphoester monomers based on COP. Functionalization of COP with polymers such as PEG or polycaprolactone results in the formation of macromonomers, which can then undergo ROP to form bottlebrush co‐polymers via a grafting‐through approach, Figure 14a. The co‐polymerization of both macromonomers further enables adjustment of overall chemical properties such as hydrophilicity [71].

**FIGURE 15:**
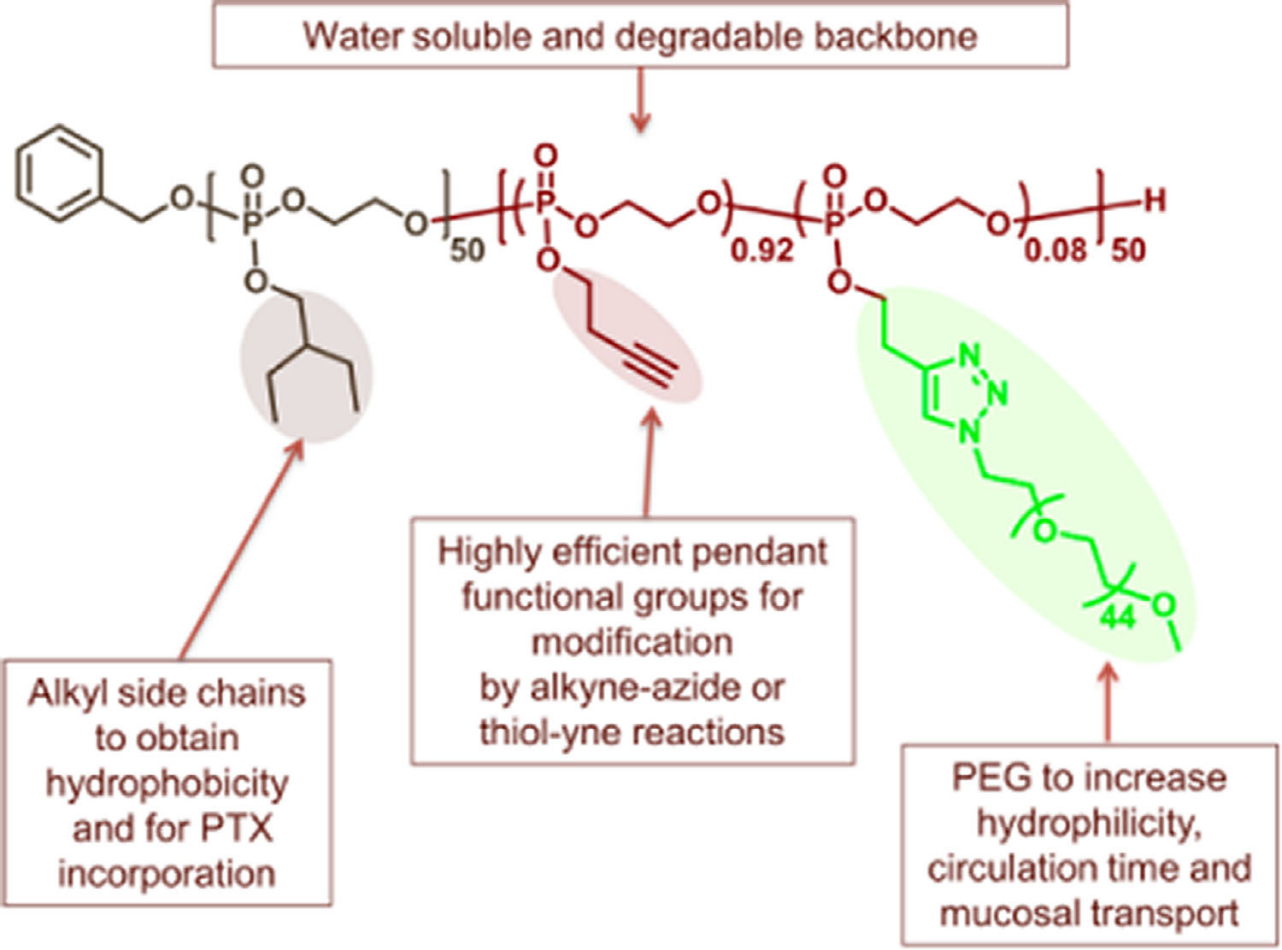
Structure of an amphiphilic PPE graft‐polymer for delivery of paclitaxel (PTX), synthesized via a grafting‐to approach through azide–alkyne Huisgen cycloaddition of PEG. Reprinted with permission from [73]. Copyright 2015 American Chemical Society.

In a similar approach, the versatility of the substitution reaction on the COP monomer can be exploited to prepare a backbone PPE bearing pendant functional groups, such as bromoisobutyrate derivatives for ATRP or alkyne‐moieties for click reactions, Figure 14b and Figure 15, respectively, enabling bottlebrush formation both via grafting‐from or grafting‐to methods [72, 73, 74]. The latter method, for example, was used for the preparation of a PPE block‐co‐polymer, which was subsequently grafted with PEG side chains, to prepare amphiphilic structures for delivery of the anticancer drug paclitaxel. Encapsulation of 10 wt% paclitaxel into these micelles or nanoparticels showed enhanced aqueous solubility of the drug, and a structure–function relationship for the drug release from these nanostructures was found. In vitro and in vivo studies provided further understanding of the effect of these architectures and their efficacy against metastatic osteosarcoma cell lines [73]. A rather unconventional synthesis pathway includes the preparation of a polyphosphite via condensation polymerization and subsequent functionalization with propargylamine in an Atherton–Todd reaction [75]. Again, the pendant alkyne functionality is available for post‐polymerization functionalization with azide‐capped PEG, forming the bottlebrush polymer.

**FIGURE 16:**
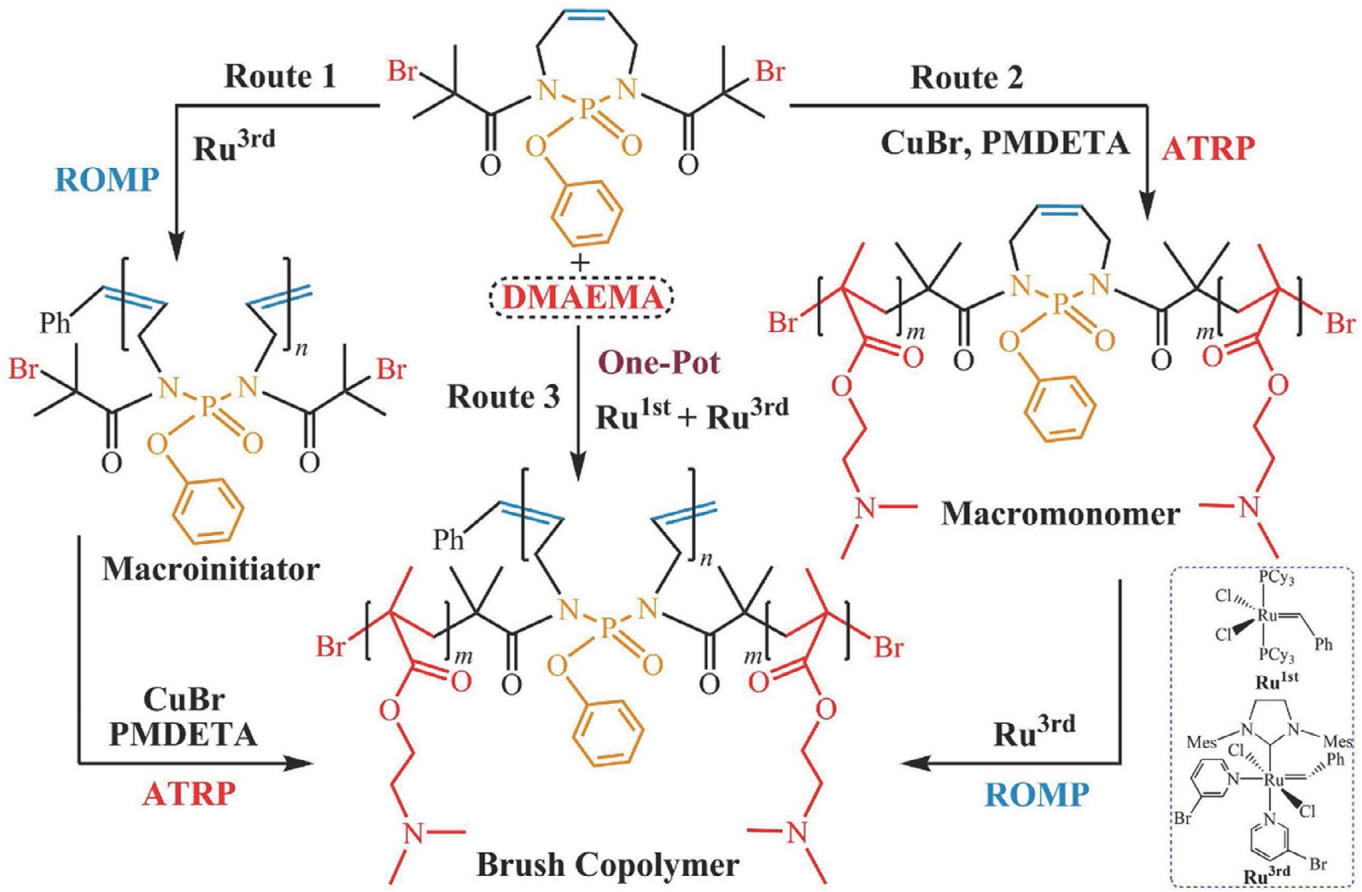
Preparation of a PPA bottlebrush co‐polymer via three different routes. Route 1: ROMP and subsequent grafting‐from of side chain polymers via ATRP; Route 2: Synthesis of a macromonomer via ATRP and subsequent grafting‐through by ROMP; Route 3: One‐pot synthesis by parallel side chain and backbone synthesis via ATRP and ROMP, respectively. Reprinted with permission from [61]. Copyright 2014 WILEY‐VCH Verlag GmbH & Co. KGaA, Weinheim.

In another innovative approach, a PPA bottlebrush polymer was reported, accessible via three different synthesis routes, Figure 16 [61]. ROMP of the cyclic phosphoramidate monomer results in the backbone, bearing two ATRP‐initiating sites per repeating unit. Subsequent grafting‐from of polymethacrylate side chains results in the final bottlebrush co‐polymer. Following Route 2, synthesis of the side chain polymers yields a macromonomer which can then be polymerized, again resulting in the bottlebrush co‐polymer. Finally, a one‐pot approach can be followed as well, yielding the final bottlebrush co‐polymer in one step.

**FIGURE 17:**
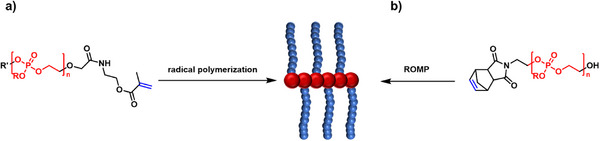
Bottlebrush polymers bearing PPE side chains prepared via grafting‐though of a ω‐chain end methacrylate functionalized PPE and free radical polymerization (a) [79] and grafting‐through of a α‐chain end norbornene functionalized PPE and ROMP (b) [80].

Bottlebrush polymers bearing PPE side chains are also accessible. For example, surface‐immobilized poly(glycidyl methacrylate) can be functionalized with tris(hydroxymethyl) aminomethane, resulting in a backbone decorated with an abundance of hydroxyl groups. These functionalities can be subsequently used for the grafting‐from of PPE side chains, allowing for tunable surface modification of silicone surfaces by careful selection of phosphoester monomers [76, 77]. Utilizing a poly(methacrylate) block‐copolymer, amphiphilic bottlebrush co‐polymers can be prepared wherein the degree of polymerization of the side‐chain PPE can be used to alter the critical aggregation concentration of the self‐assembling aggregates [78]. To provide bottlebrush polymers with PPE side‐chains via a grafting‐through approach, the PPE macromonomers have to be prepared either by α‐or ω‐end group functionalization, Figure 17. On the one hand, end‐capping a PPE with 2‐isocyanatoethyl methacrylate results in a macromonomer for grafting‐through by free radical polymerization [79]. On the other hand, a norbornene derivative bearing an alcohol group can be used as an initiating species for the ROP of PPE and subsequently polymerized via ROMP [80].

#### 3.2.3 Star‐Branched Polymers

**FIGURE 18:**
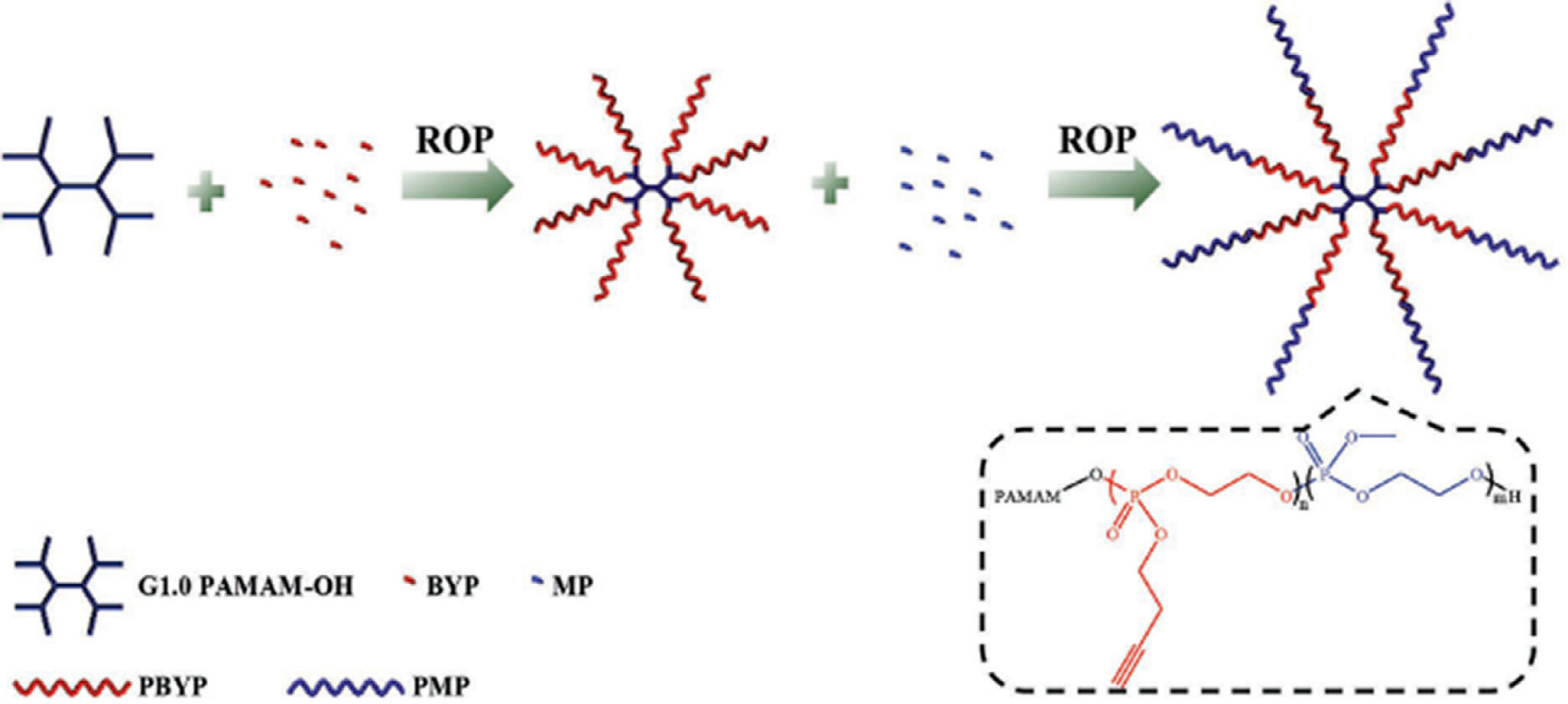
Schematic synthesis pathway toward an 8‐arm poly(amidoamine)‐block‐poly(2‐­butynyl phospholane)‐block‐poly(2‐methoxy phospholane) star‐shaped block‐copolymer (PAMAM‐PBYP‐PMP) for use as a micellar drug delivery agent. Reprinted with permission from [81]. Copyright 2017 WILEY‐VCH Verlag GmbH & Co. KGaA, Weinheim.

Literature regarding star‐branched phosphate‐based polymers is less frequent than bottlebrush polymers or in contrast to star‐branched PPz. Nevertheless, various systems are reported, often using a divergent approach via a hydroxyl‐functionalized core‐molecule as initiator for ROP of cyclic monomer derivatives. For example, a hydroxyl terminated poly(amido amine) dendrimer of the first generation was used as a core structure for an 8‐arm star‐branched co‐polymer, Figure 18 [81]. After sequential ROP of 2‐butynyl phospholane (BYP) and 2‐methoxy phospholane (MP), the resulting 8‐armed star‐shaped amphiphilic copolymer poly(amidoamine)‐block‐poly(2­butynyl phospholane)‐block‐poly(2‐methoxy phospholane) was investigated for its self‐assembling behavior, as well as for drug delivery. High drug encapsulation efficiency of doxorubicin up to 90% into the micelles and excellent anticancer activity toward HeLa cells were reported, as well as sustained drug release in a simulated intracellular lysosome environment [81]. 4‐arm star‐shaped co‐polymers of poly(ethyl ethylenephosphate) with organic polyesters such as poly(d,l‐lactide) [82] or poly(ε‐caprolactone) [83] have been reported. Again, the amphiphilic character of the block co‐polymer allows for self‐assembly and micelle formation for potential use as drug delivery vehicles.

**FIGURE 19:**
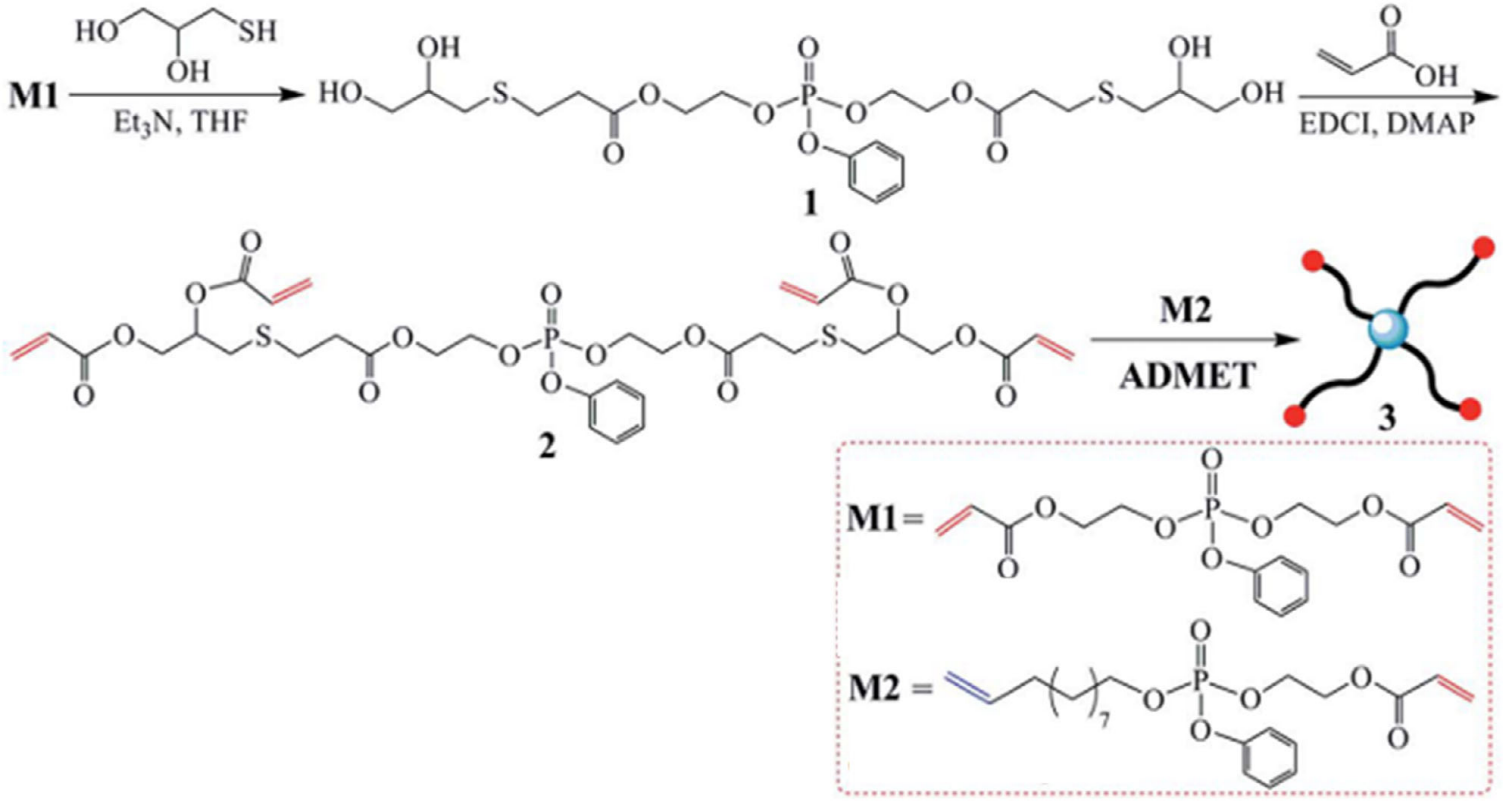
Synthesis of a star‐shaped PPE via ADMET polymerization. Reproduced from [64] with permission from the Royal Society of Chemistry.

A convergent method for the preparation of star‐branched PPE depends on ADMET polymerization [64]. For this method, an asymmetric α,ω‐diene phosphoester monomer, **M2** in Figure 19, containing a terminal double bond and an acrylate, was polymerized in a head‐to‐tail manner. In parallel, a multifunctional chain‐stopper bearing four acrylate end‐groups was synthesized, structure **2** in Figure 19, and successfully utilized as the core structure for the 4‐arm star‐branched PPE.

**FIGURE 20:**
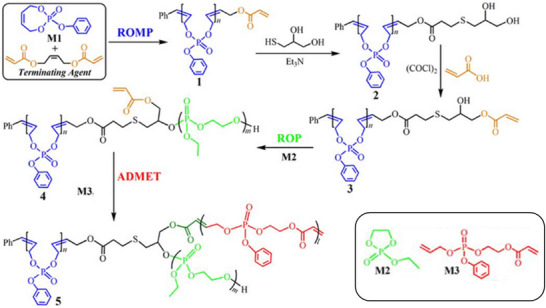
Synthetic pathway toward an ABC miktoarm 3‐arm star PPE via a sequential combination of ROMP, ROP, and ADMET polymerization. Reprinted with permission from [63]. Copyright 2015 Wiley Periodicals, Inc.

By strategically combining ROMP, ROP, and ADMET polymerization, even ABC miktoarm 3‐arm star PPEs are feasible [63]. The synthetic route is illustrated in Figure 20 and sequentially utilizes the different polymerization methods. After preparation of the first arm via ROMP, the growing chain is terminated with an acrylate group. This functional end‐group is, in turn, modified toward a diol through a thiol‐ene reaction with 1‐thioglycerol and controlled mono‐esterfication with acrylic acid. Ring‐opening polymerization of ethyl ethylene phosphate from the free hydroxyl group serves as an AB‐block copolymer, bearing the remaining acrylate functionality at the co‐polymer junction. Finally, the third arm of the star‐branched polymer was synthesized separately via ADMET polymerization of an asymmetric α,ω‐diene phosphoester monomer and incorporated into the complete structure through an end‐capping reaction with the AB‐block co‐polymer. Despite the synthetic complexity of the approach, it remarkably showcases the versatility and potential of inorganic polymers in macromolecular architectural design.

### 3.3 Phosphorus‐Based Dendrimers

**FIGURE 21:**
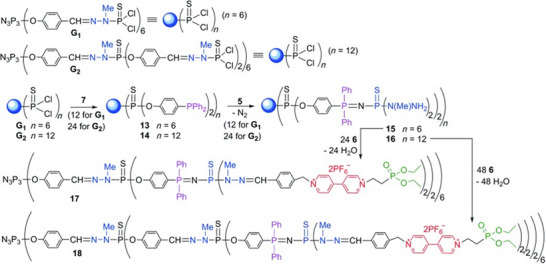
Synthesis pathway toward generation 2 and generation 3 “onion‐peel” phosphorus‐dendrimers, containing six and seven different phosphorus species, respectively. Reprinted with permission from [91]. Copyright 2015 WILEY‐VCH Verlag GmbH & Co. KGaA, Weinheim.

Phosphorus‐based dendrimers incorporate phosphorus as a structural building block, predominantly at their branching sites, resulting in unique architectural possibilities and diverse accessible chemistry [84, 85, 86, 87]. In one of the first reports on phosphorus dendrimers, nucleic acid phosphoramidates were used as monomeric units, a thymine derivative for chain extension, and an adenosine derivative as a branching point. Interestingly, the approach remains one of the few examples of a convergently synthesized phosphorus‐based dendrimer. Additionally, through the application of an automated DNA synthesizer for the preparation of the dendrimers in the solid phase, the major drawback of the complex iterative synthesis pathway of dendrimers could be mitigated [88]. In the more common divergent approach, the dendrimers are built up starting from their core, for which hexachlorotriphosphazene or thiophosphoryltrochloride are mostly used [89]. In general, the chemistry of such structures has already been established for several decades, comprising the substitution of the chlorine atoms in the first step and subsequent formation of the following generations through condensation reactions with N‐methyldichlorothiophosphorhydrazide or Staudinger reactions with an azidothiobishydrazinophosphine derivative, for example [90]. Nevertheless, the versatile phosphorus‐chemistry and the combination of the various approaches result in a diverse toolbox for the construction of different dendrimer architectures. This diversity was remarkably highlighted in an approach in which dendritic structures containing up to seven distinct phosphorus species were prepared, Figure 21. These so‐called “onion peel” phosphorus nanodendritic systems enable a selective ligation of Au(I) via the presence of aurophilic P═N─P═S moieties and were investigated for medical and material applications [91].

**FIGURE 22:**
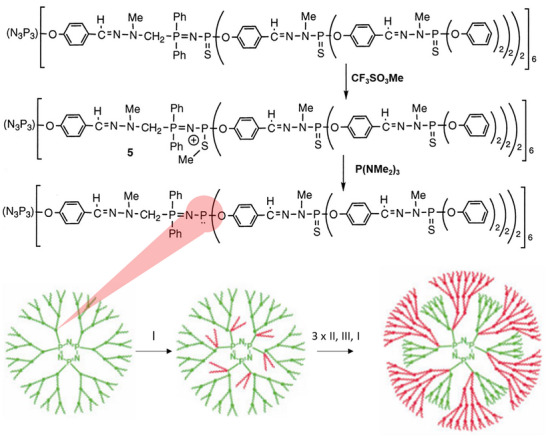
Stepwise functionalization of a phosphorus‐based dendrimer and subsequent formation of internal dendritic units inside the void space of the original dendrimer. I: N_3_P(S)(OC_6_H_4_CHO)_2_; II: H_2_NNMeH; III: Ph_2_OCH_2_OH. From [92]. Reprinted with permission from AAAS. Copyright 1997 The American Association for the Advancement of Science.

Aside from its aurophilic character, the presence of the P═N─P═S fragment can be exploited for architectural means as well. Selective alkylation with methyltrifluoromethane sulfonate results in the formation of a cationic P═N─P─S─Me species, which undergoes a clean S─Me transfer upon reaction with tris(dimethylamino)phosphane and yields a highly reactive P═N─P fragment, Figure 22 [92]. A Staudinger reaction with a phosphorazidothioate opens up a new branching point in the void space of the original dendrimer, which was further grown to internal dendrimers up to generation 4.

**FIGURE 23:**
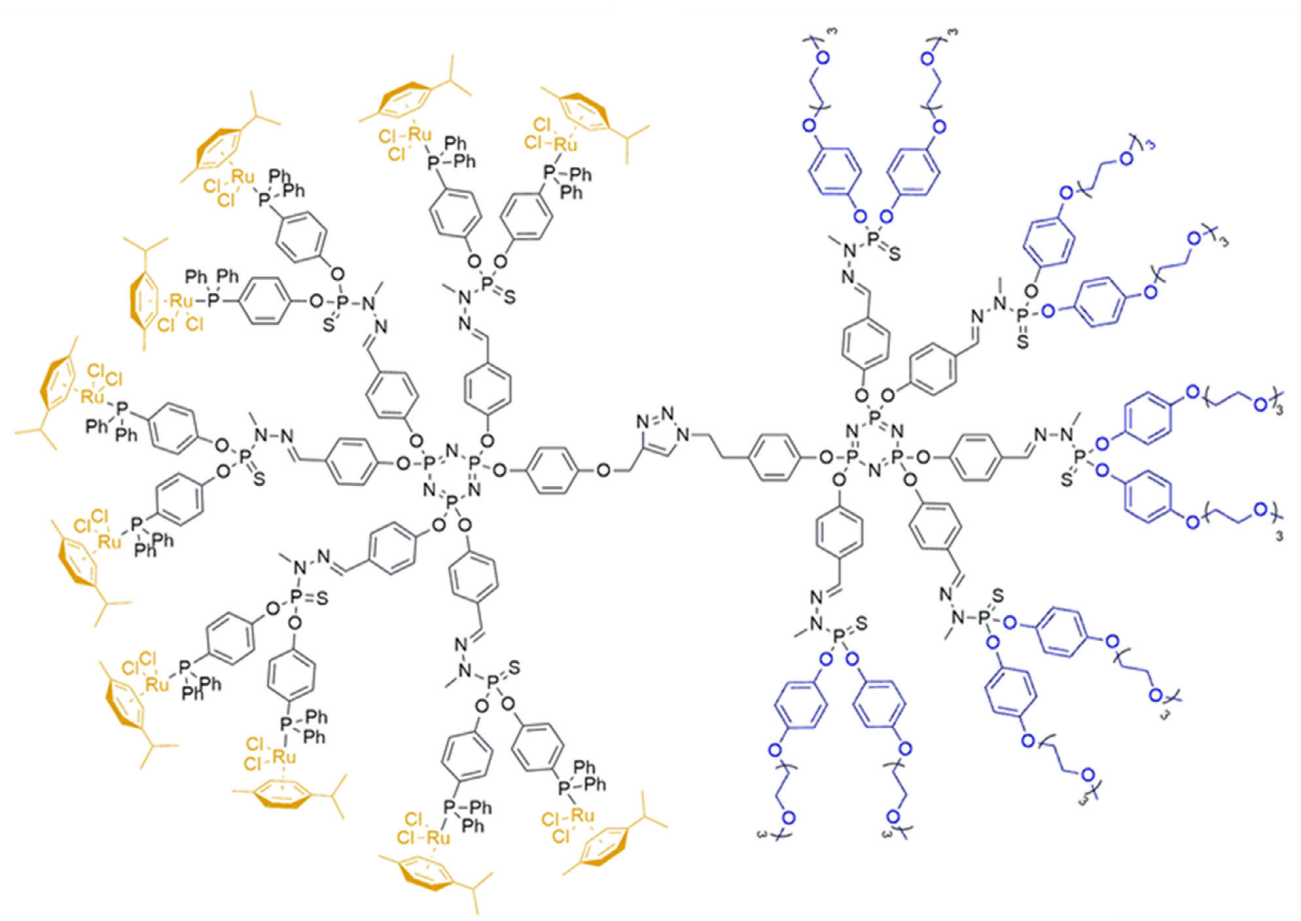
Structure of a phosphorus‐based Janus dendrimer for application in catalysis. Reproduced from [93] with permission from the Royal Society of Chemistry. Licensed under CC BY‐NC 3.0.

In another approach, a Janus dendrimer was prepared after asymmetrically substituting the hexchlorotriphosphazene core with a single azide or acetylene group, along with the conventional 4‐hydroxybenzaldehyde [93]. The azide‐bearing AB_5_‐dendron was first PEGylated, and the resulting G_1_‐PEG was subsequently coupled to the acetylene‐bearing G_0_‐dendron. Finally, the G_0_‐dendron half was reacted with N‐methyldichlorothiophosphorhydrazide and (4‐(diphenylphosphoryl)phenol, enabling immobilization of a Ru‐complex on the Janus dendrimer and its application as a recyclable catalytic system, Figure 23.

**FIGURE 24:**
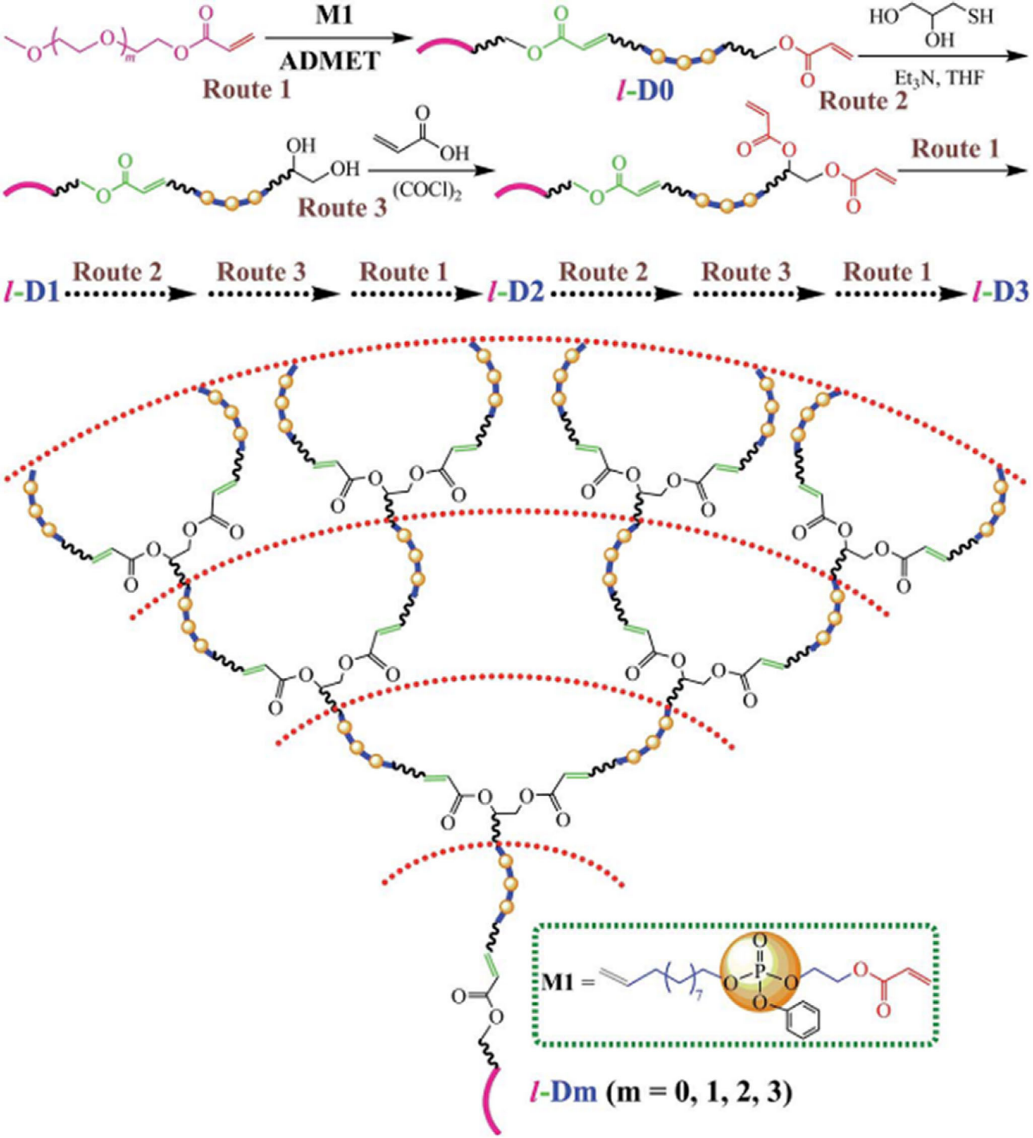
Synthesis of a linear‐dendron PPE via ADMET polymerization. Reproduced from [94] with permission from the Royal Society of Chemistry.

Analogous to the formation of the star‐branched polymers described in Figure 19, linear‐dendron PPEs have also been synthesized [94]. A linear PPE block co‐polymer was first obtained via ADMET polymerization of the asymmetric α,ω‐diene phosphoester monomer **M1**, Figure 24, and reacted into the chain‐end functionalized di‐acrylate. However, unlike most dendrimers, where growth occurs from the end groups, this architecture is constructed by end‐capping separately polymerized PPE chains with a dendritic end‐capper.

## 4 Silicon‐Based Architectures

**FIGURE 25:**
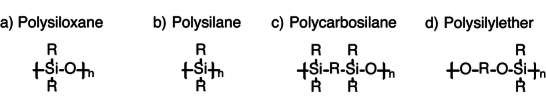
Main types of silicon‐based polymers.

Silicon‐based polymers are characterized by their chemical and structural versatility, offering a robust platform for architectural design, similar to the phosphorus‐based polymers described in the previous chapter. Their backbones exhibit diverse chemical frameworks, Figure 25, forming the foundation of their material properties, which can be further fine‐tuned through strategic selection of incorporated building blocks [36, 95]. Among these, polysiloxanes, including the widely used PDMS, are well known for their high flexibility, thermal stability, and biocompatibility, making them highly attractive for biomedical applications. Polysilylethers (PSE), in contrast, offer enhanced hydrolytic degradability, expanding their relevance in transient or resorbable medical materials [96]. Additionally, polysilanes and polycarbosilanes, which feature Si–Si or Si–C backbones, respectively, contribute unique properties such as electronic conductivity or thermal resistance and are under investigation for use in electronic materials and high‐performance ceramics. Overall, the high stability of silicon‐based backbones combined with their tunable reactivity renders them valuable not only in technical applications but also across a range of biomedical contexts.

### 4.1 Bottlebrush Architectures

**FIGURE 26:**
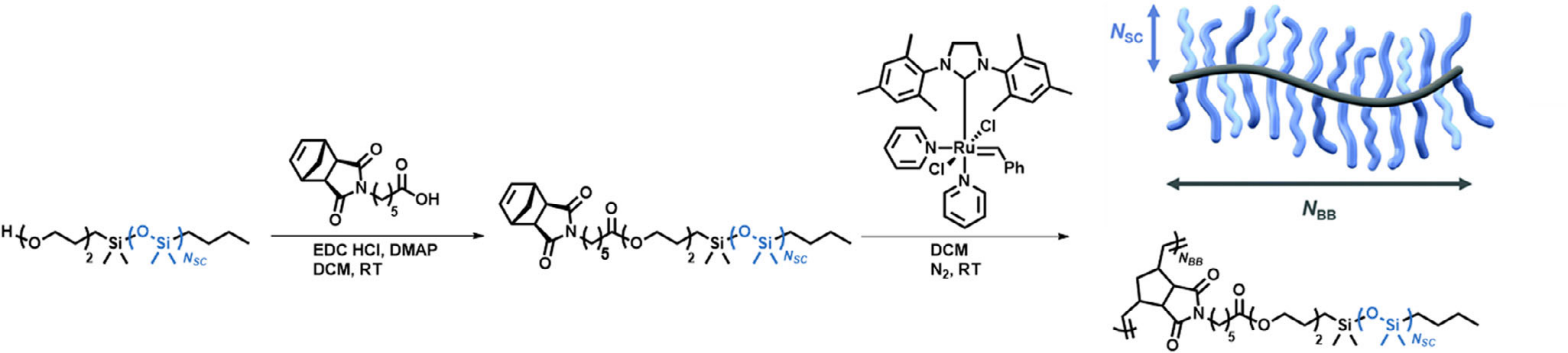
Schematic representation of the synthesis pathway for bottlebrush polymers using ROMP. Reproduced from [98] with permission from the Royal Society of Chemistry.

A prominent example of macromolecular design of silicon‐based polymers is bottlebrush architectures. Herein, the ROMP method is highlighted as one of the most precise strategies in this field, polymerizing a norbornene‐derivatized macromonomer in a grafting‐through approach using a Grubbs catalyst, which enables excellent control over dispersity and polymer length [97]. This method was used, for example, for the synthesis of bottlebrush polymers for pressure‐sensitive applications, including capacitive pressure sensors. The synthesis involves the preparation of PDMS macromonomers by functionalizing commercially available PDMS with a norbornene end‐group via esterification and subsequent ROMP to form the bottlebrush architecture, Figure 26 [98]. Crosslinking can be achieved using a synthesized PDMS‐based benzophenone crosslinker, which allows precise control over the network structure and mechanical properties of the material. These elastomers exhibited lower shear moduli (10–100 kPa) and a 53‐fold increase in sensitivity compared to conventional linear elastomers like Sylgard 184. This cross‐linking approach was further developed into a universal strategy for bottlebrush polymer networks in bulk and thin‐film systems [99].

**FIGURE 27:**
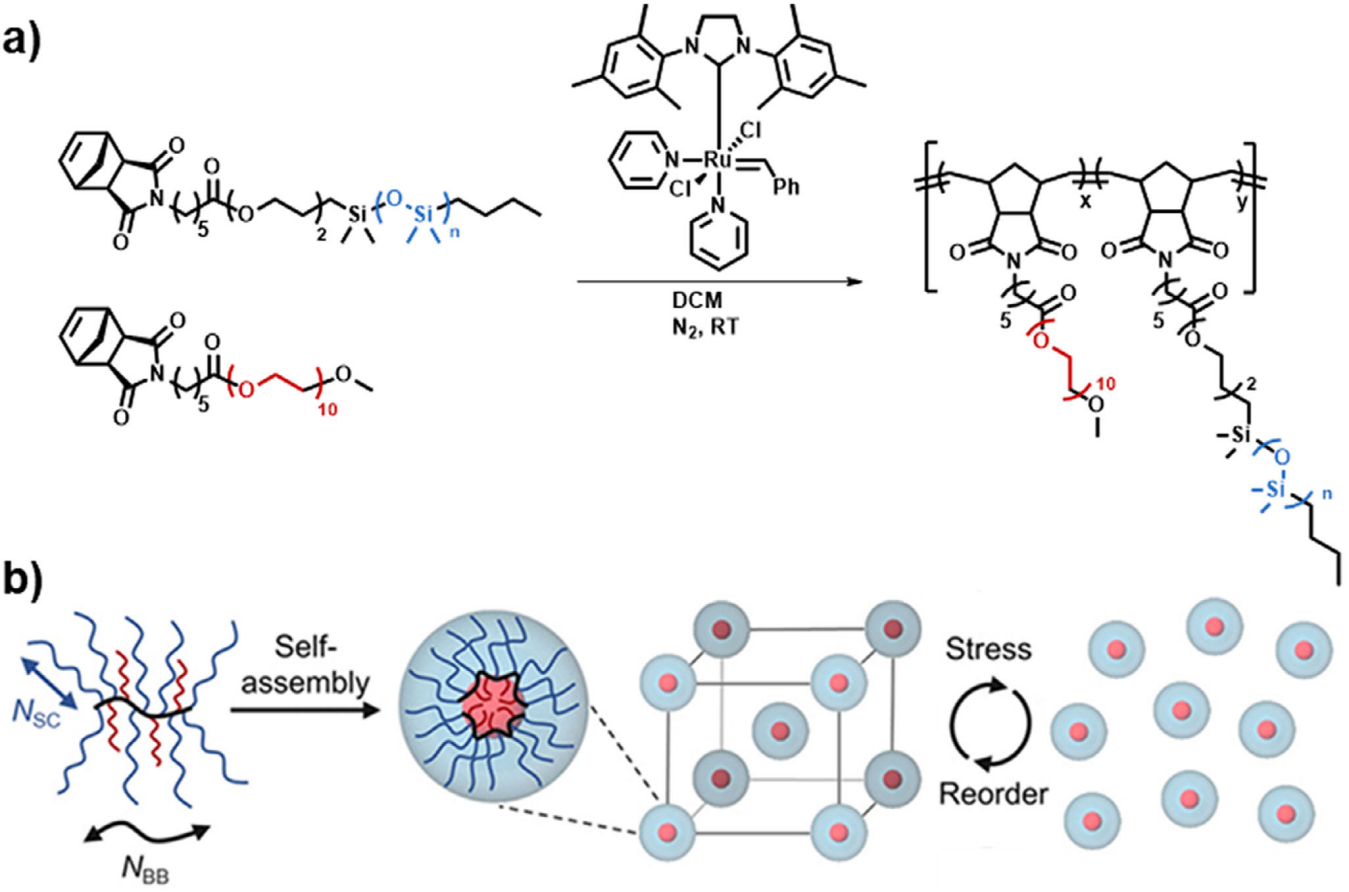
(a) Synthetic route for PDMS‐stat‐PEG bottlebrush copolymers via ROMP, utilizing a norbornene‐functionalized polysiloxane and poly(ethylene glycol) macromonomer. (b) Schematic representation of the self‐assembly process into a body‐centered cubic nanostructure, enabling stress‐induced reordering and solvent‐free processing for scalable applications. From [100]. Reprinted with permission from AAAS. Licensed under CC BY‐NC 4.0.

The simple synthesis of the norbornene‐macromonomers additionally expedites the preparation of co‐polymers. PEG co‐macromonomers were incorporated into this system via copolymerization of norbornene‐functionalized PEG, Figure 27. The resulting statistical bottlebrush copolymers self‐assembled into a body‐centered cubic spherical phase, enabling a reversible shear‐induced phase transition. Under shear stress, the material exhibits fluid‐like behavior and returns to its original solid state after stress release, facilitating solvent‐free 3D printing [100]. The precise architectural control of these polymers directed their self‐assembly into well‐defined nanostructures with distinct long‐range order, including spherical and cylindrical morphologies, where PDMS, due to its low glass transition temperatures, contributed to high structural stability and slow defect relaxation [101].

**FIGURE 28:**
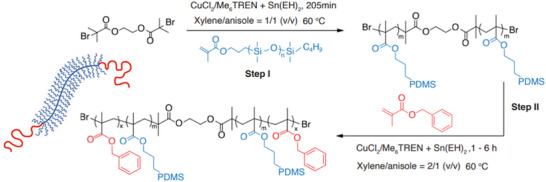
Schematic representation of the two‐step ATRP process used to synthesize triblock linear‐bottlebrush‐linear copolymers. Adapted with permission from [104]. Copyright 2021 American Chemical Society.

Based on the same underlying principle, bottlebrush polymer networks were synthesized by self‐assembling linear‐bottlebrush‐linear triblock copolymers [102, 103, 104]. The triblock copolymers were prepared based on a bifunctional ATRP‐initiator, first polymerizing a commercially available methacrylate functionalized PDMS to form a densely packed bottlebrush. Based on the living chain ends, the PDMS‐bottlebrush was further used as a difunctional macroinitiator, forming linear end blocks as shown in Figure 28. In their work, they demonstrated how macromolecular architecture influences domain morphologies and how such systems could be utilized in additive manufacturing, as well as explored the dynamic mechanical properties of the resulting networks. Based on a similar architecture but different synthetic strategy, polystyrene‐PDMS‐polystyrene linear‐bottlebrush‐linear triblock co‐polymers have been synthesized via a “grafting onto” strategy using well‐defined anionic precursors. Unlike the “grafting through” approach, this method allows separate synthesis and characterization of backbone and side chains, enabling precise architectural control and consistent properties. The group showed that these copolymers form soft thermoplastic elastomers through microphase separation, with tunable mechanics governed by grafting density and block lengths [105]. In a follow‐up study, a 3D‐printable, solvent‐free elastomer was developed by combining polystyrene‐PDMS diblocks with polystyrene‐PDMS‐polystyrene triblocks. The architecture supports direct ink writing without post‐treatment, with yield stress and recovery behavior arising from the controlled self‐assembly of bottlebrush networks [106].

**FIGURE 29:**
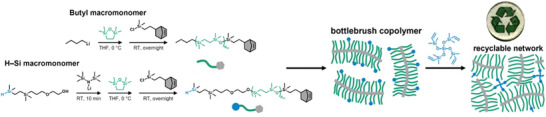
Synthesis of bottlebrush copolymers via ring‐opening metathesis copolymerization and network formation via hydrosilylation, resulting in a recyclable elastomer. Reprinted from [107], licensed under CC BY NC ND 4.0.

In another interesting approach, poly(2,2,5,5‐tetramethyl‐2,5‐disila‐1‐oxacyclopentane) (PTMOSC) was employed as the side chain polymer, emphasizing the versatility of inorganic polymers by providing a recyclable system [107]. The synthesis began with the anionic ring‐opening polymerization of a cyclic carbosiloxane monomer to generate PTMOSC‐macromonomers end‐capped with norbornene chlorosilane. Polymerization was initiated using either n‐butyllithium or a previously reported H─Si─O initiator [108], Figure 29. The monofunctional and heterotelechelic macromonomers were subsequently polymerized into functionalized bottlebrush architectures. Super‐soft polymer networks with tunable mechanical properties (moduli ranging from 3 to 40 kPa) were further created through a hydrosilylation reaction with a four‐arm crosslinker, as shown in Figure 29. These networks were fully recyclable via hydrolysis of the siloxane bonds, recovering over 85% of the original PTMOSC‐monomer.

**FIGURE 30:**
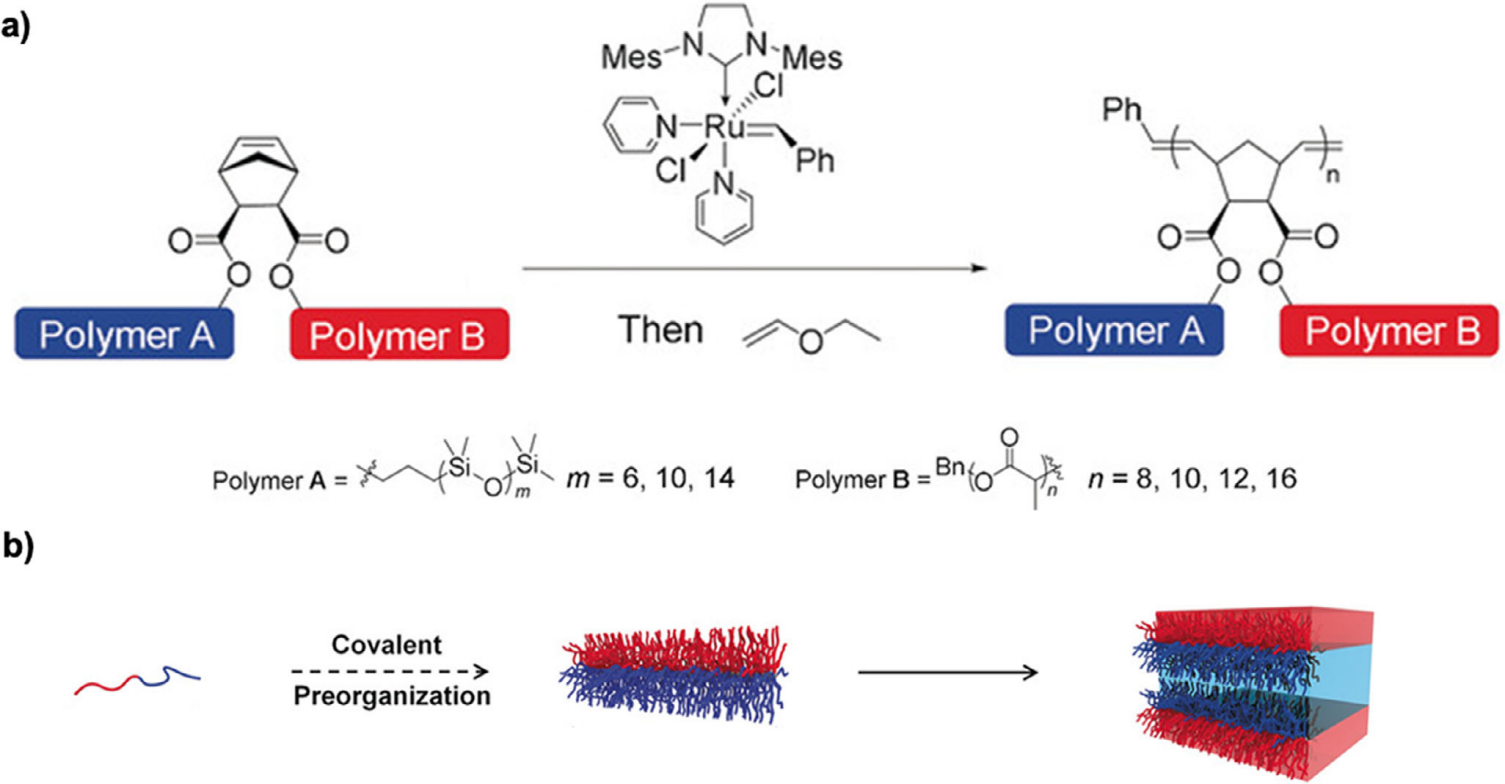
(a) Schematic representation of the synthesis of Janus graft block copolymers. (b) Idealized illustration of the self‐assembly of the Janus graft block copolymers. Reprinted with permission from [109]. Copyright 2018 Wiley‐VCH Verlag GmbH & Co. KGaA, Weinheim.

The norbornene framework can also be applied for the preparation of Janus graft block copolymers with pseudo‐alternating side chains of PDMS and polylactide, Figure 30 [109]. The precise structure is achieved through ROMP of norbornene macromonomers, containing both side chain polymers, and this design enables the formation of highly ordered nanostructures with exceptionally small domains down to 2.8 nm. In addition, properties such as the nanodomain size of the self‐assembly or thermomechanical properties of the system can be independently tuned based on the side‐chain and backbone characteristics, respectively, effectively decoupling polymer properties from nanostructure characteristics.

**FIGURE 31:**
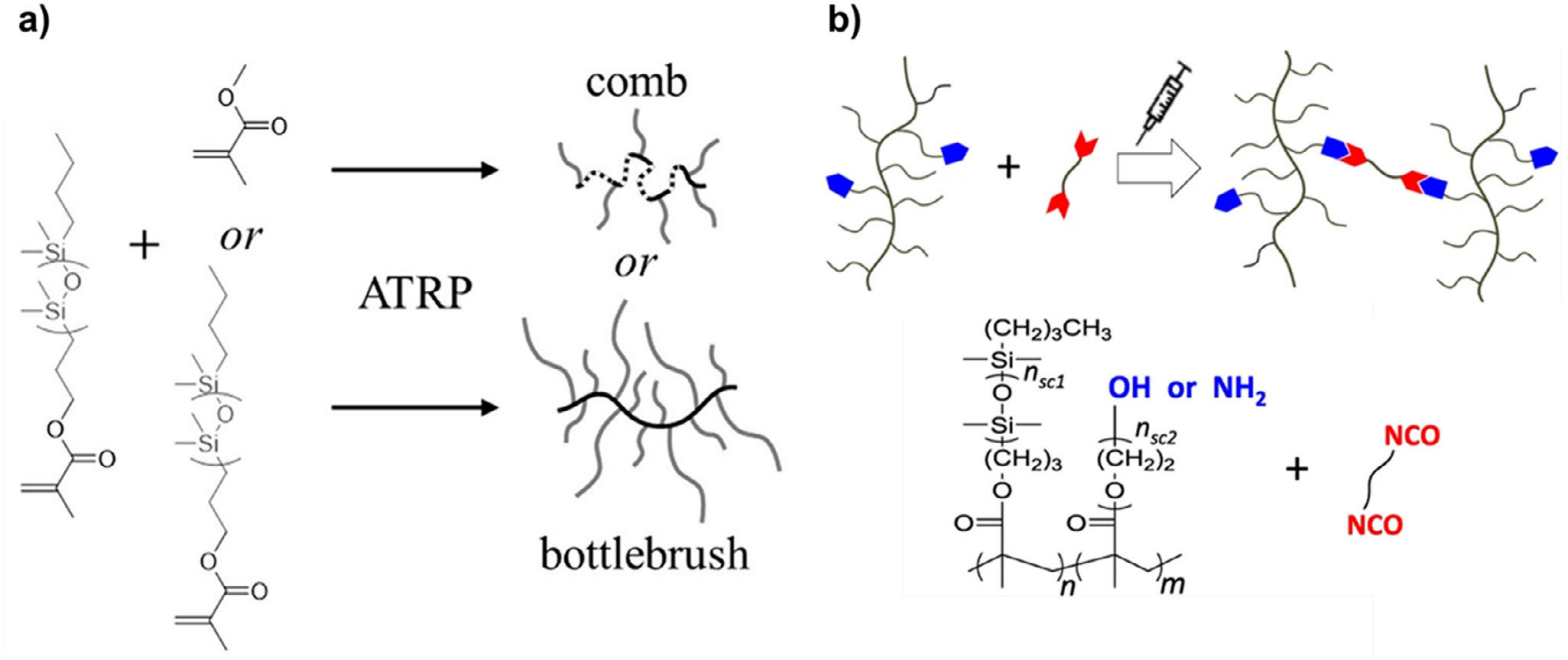
Synthesis of PDMS comb and bottlebrush polymer architectures via ATRP of acrylate‐capped PDMS side chains (a) [112] and crosslinking via reactive side‐chain functionalization (b) [25]. Adapted with permission from [112]. Copyright 2020 American Chemical Society. Reprinted from [25], licensed under CC BY 4.0.

The research group led by Sheiko et al. extensively investigated bottlebrush polymers with commercially available PDMS acrylate side chains, employing a grafting‐through approach via ATRP. This method enabled the direct incorporation of PDMS macromonomers into the polymer backbone, resulting in a high grafting density and precise architectural control (Figure 31a) [22, 110, 111, 112, 113]. One example incorporates hydroxyl‐functionalized poly(ethylene glycol) methacrylate (PEGMA) co‐macromonomers, allowing increased control over reactivity and crosslinking behavior. The hydroxyl groups of PEGMA served as reactive sites for further chemical modifications, including the introduction of azides, amines, furan moieties, or acrylate end groups [25]. By systematically adjusting the proportion of functionalized side chain ends and fine‐tuning the polymer backbone, the mechanical properties of injectable, non‐leaching elastomers could be optimized (Figure 31b). This work demonstrated that such PDMS‐based bottlebrush elastomers can be directly injected in vivo and cured into tissue‐mimetic implants with tunable mechanics (1–100 kPa), thereby covering the elasticity range of many soft tissues such as brain, fat, and muscle. Importantly, the materials exhibited high biocompatibility with minimal fibrotic encapsulation and stable integration into surrounding tissue over several months. Their non‐leaching character, combined with the ability to finely adjust viscoelastic properties via side chain chemistry, underscores their promise as customizable soft implants. These findings highlight the potential of PDMS‐based bottlebrush systems in reconstructive surgery, regenerative medicine, and other areas where mechanically adaptive, long‐term stable biomaterials are required. In a related development, PDMS‐based bottlebrush elastomers have also been engineered with electrical conductivity, yielding ultrasoft materials (< 11 kPa) suitable for skin‐conformal biointerfaces and potentially for neural applications [113].

**FIGURE 32:**
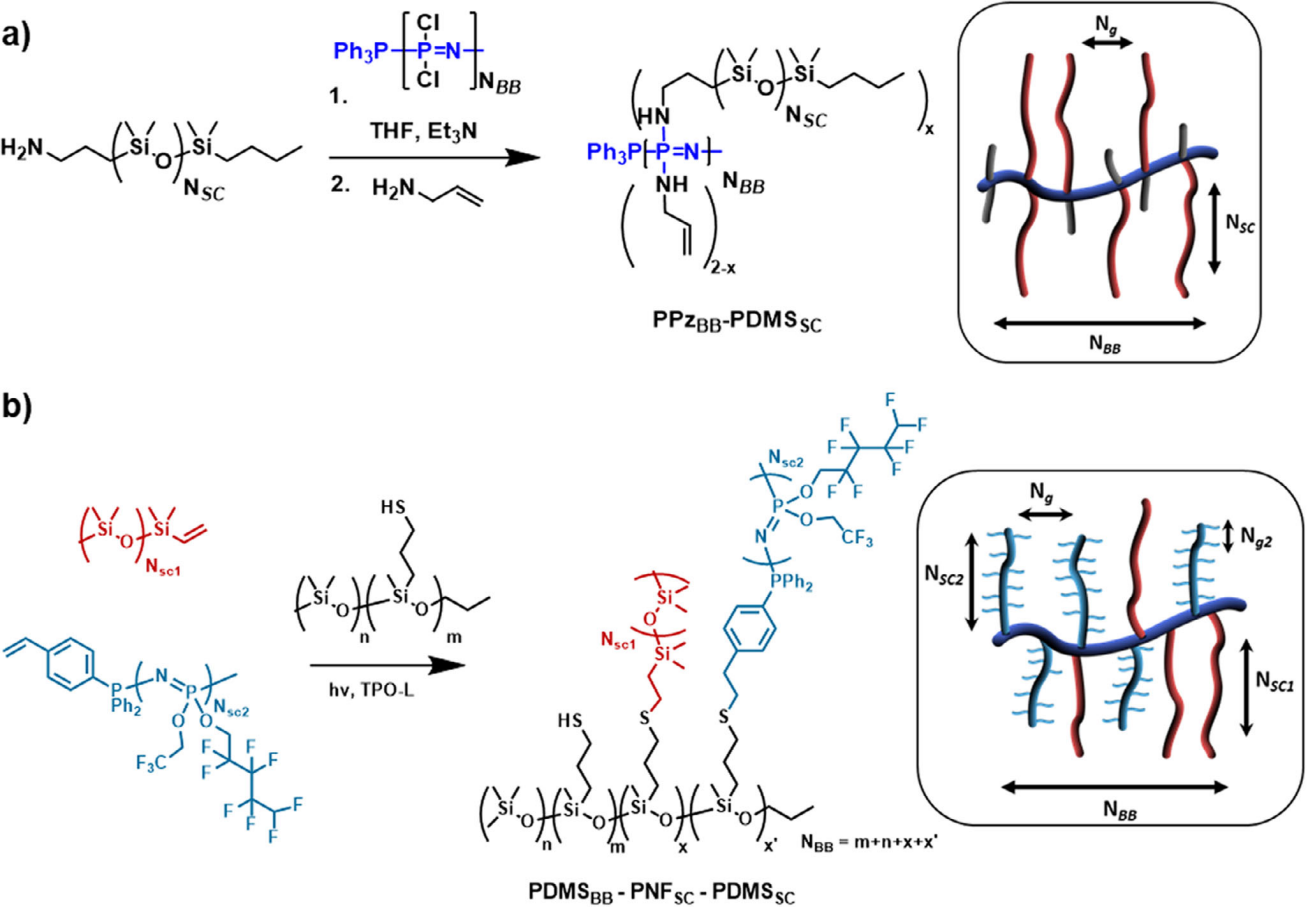
Schematic synthesis path toward PPz‐PDMS bottlebrush polymers and their architectural characteristics, in turn, tightly controlled through the synthetic approach. (a) PPz‐g‐PDMS bottlebrush polymers via a grafting‐to approach, facilitated by the macromolecular substitution reaction of PPz. (b) PDMS‐g‐PPz bottlebrush polymers via a grafting‐to approach, facilitated by thiol‐ene addition‐chemistry. Reprinted from [24], licensed under CC BY 4.0.

A fully inorganic, siloxane bottlebrush material was prepared by synthesizing a polymethylhydrosiloxane through cationic equilibrium ROP and subsequent chlorination into polychloromethylsiloxane, yielding a functionalized backbone structure [114]. The PDMS side chains were polymerized by *tert*‐butyl lithium‐initiated anionic ROP, and the reactive chain‐ends were subsequently grafted onto the backbone, substituting the chlorine atoms. The architecture could be highly controlled, with dispersities of Đ ≈ 1.1, and the PDMS‐*g*‐PDMS bottlebrush systems were extensively investigated [114, 115, 116, 117, 118]. Recently, the synthesis of inorganic bottlebrush polymers combining PPz and PDMS was reported by our group, highlighting the unique interplay between these two polymer classes [24]. A PPz backbone, prepared via phosphine‐mediated living cationic polymerization, was subsequently functionalized with mono‐amine‐terminated PDMS side chains in a grafting‐to approach (Figure 32a). In a complementary strategy, PDMS backbones were modified with pendant thiol groups and reacted with vinyl‐terminated PPz via thiol–ene chemistry to yield bottlebrush structures with reversed composition (Figure 32b). As already mentioned above, especially the former system enables unprecedented grafting densities, given the two side chains per PPz‐repeating unit. A key aspect of this work was the systematic investigation of grafting density and macromolecular design, which allowed for precise control over the polymer's properties and facilitated the development of materials with tailored architectures and enhanced functionality.

**FIGURE 33:**
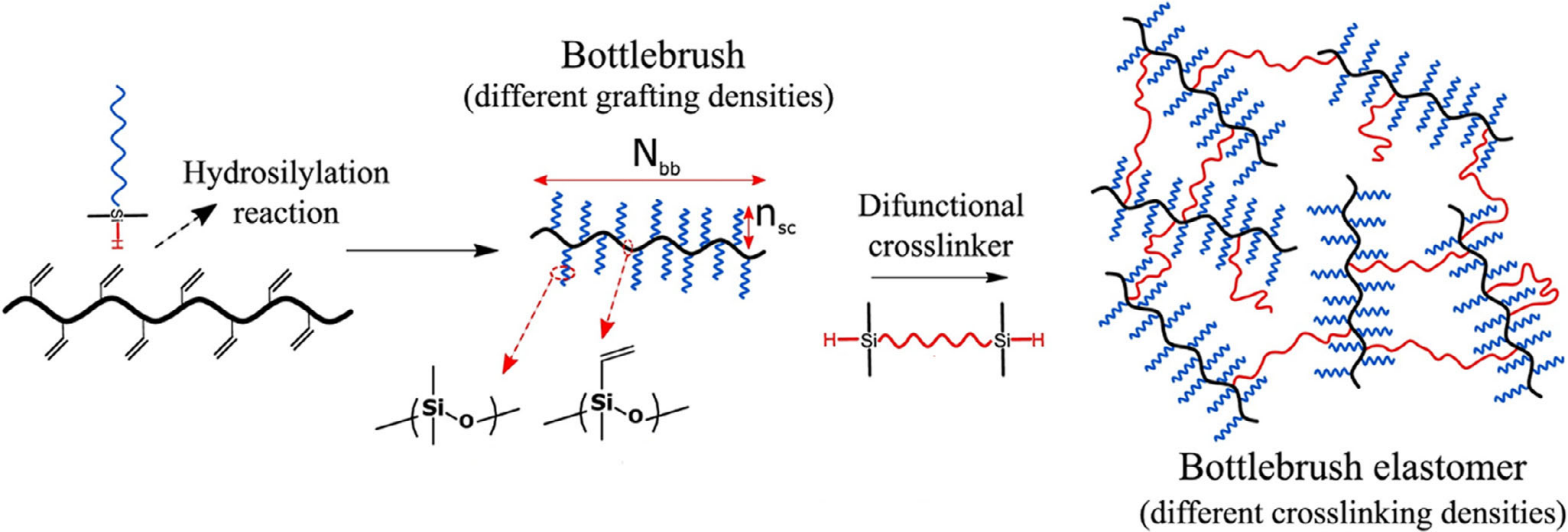
Bottlebrush polymers are synthesized via hydrosilylation, where hydride‐terminated side chains are grafted onto a vinyl‐functionalized backbone, followed by crosslinking with siloxane linkers to achieve tunable network densities. Reprinted from [119], licensed under CC BY 4.0.

Due to its high efficiency, chemoselectivity, and compatibility with PDMS‐based systems, hydrosilylation reactions have become a fundamental tool for designing tailored macromolecular architectures. These polymers are prepared via a grafting‐to approach, where hydride‐terminated side chains are covalently attached to a vinyl‐functionalized backbone in a controlled manner, as shown in Figure 33 [119], or vice versa. A representative example of the latter was reported by Hu et al., who used multi‐hydride‐functional PDMS as the backbone and mono‐vinyl‐terminated PDMS as grafts to generate bottlebrush elastomers via platinum‐catalyzed hydrosilylation [120]. For example, a one‐step hydrosilylation and crosslinking process eliminates entanglements in bottlebrush architectures and enables the fabrication of crosslinked networks with tunable elastic moduli ranging from 1 to 100 kPa. Demonstrating the versatility of this method for developing soft, solvent‐free materials [121, 122]. Additionally, the incorporation of these bottlebrush systems into dynamic double‐network structures allows for high‐molecular‐weight architectures with exceptional properties, including self‐healing and energy absorption [123].

**FIGURE 34:**
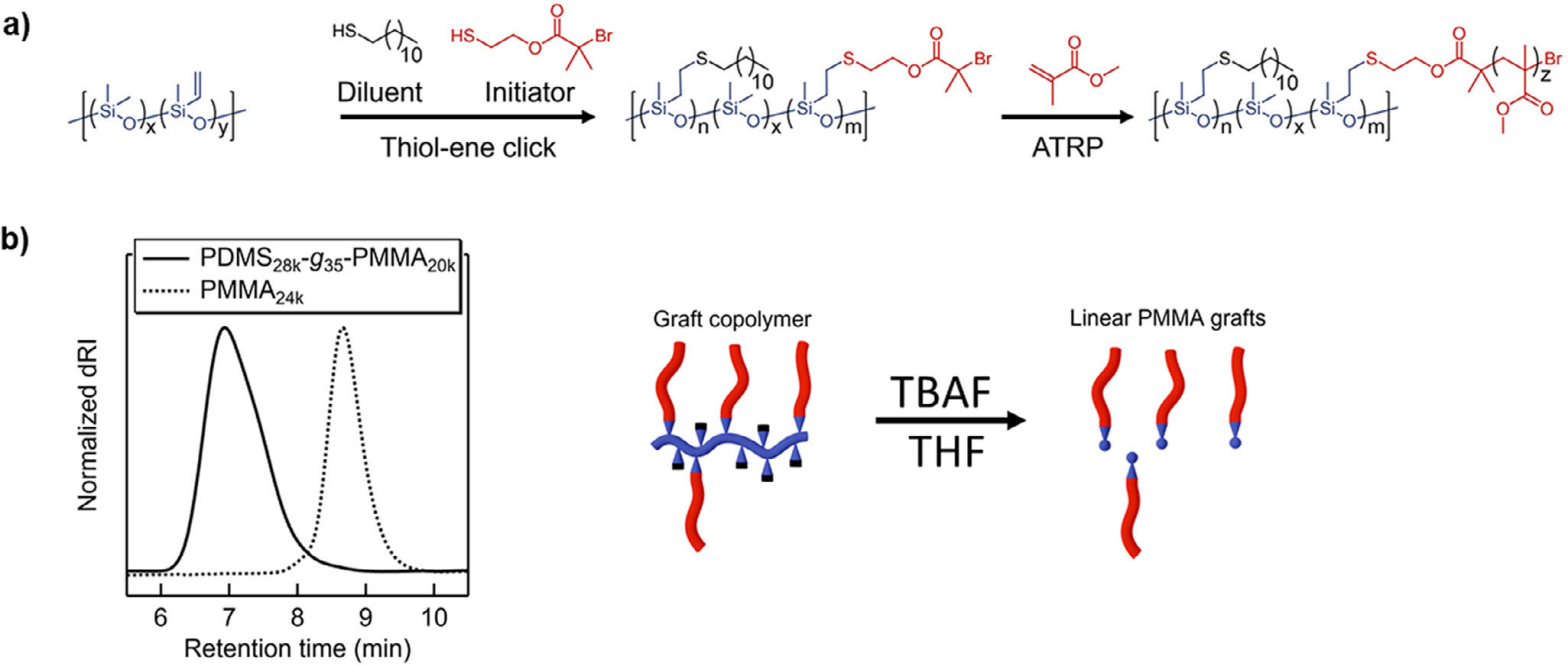
(a) Two‐step synthesis of siloxane‐based graft copolymers via a thiol‐ene addition reaction and subsequent ATRP. (b) SEC trace and schematic illustrating backbone cleavage using TBAF to release linear poly(methyl methacrylate) (PMMA) grafts. Adapted with permission from [124]. Copyright 2023 The Authors. Journal of Polymer Science published by Wiley Periodicals LLC.

In a grafting‐from route, a PDMS‐backbone bearing pendant vinyl‐groups was functionalized via a thiol‐ene addition‐reaction with an ATRP‐initiator moiety, Figure 34 [124]. Control over the grafting density was governed by the addition of 1‐dodecanethiol as a diluent. The controlled character of the grafting reaction was verified after selective degradation of the PDMS backbone using tetrabutylammonium fluoride (TBAF) and analysis of the side chains through size exclusion chromatography.

### 4.2 Star‐Branched Architectures

**FIGURE 35:**
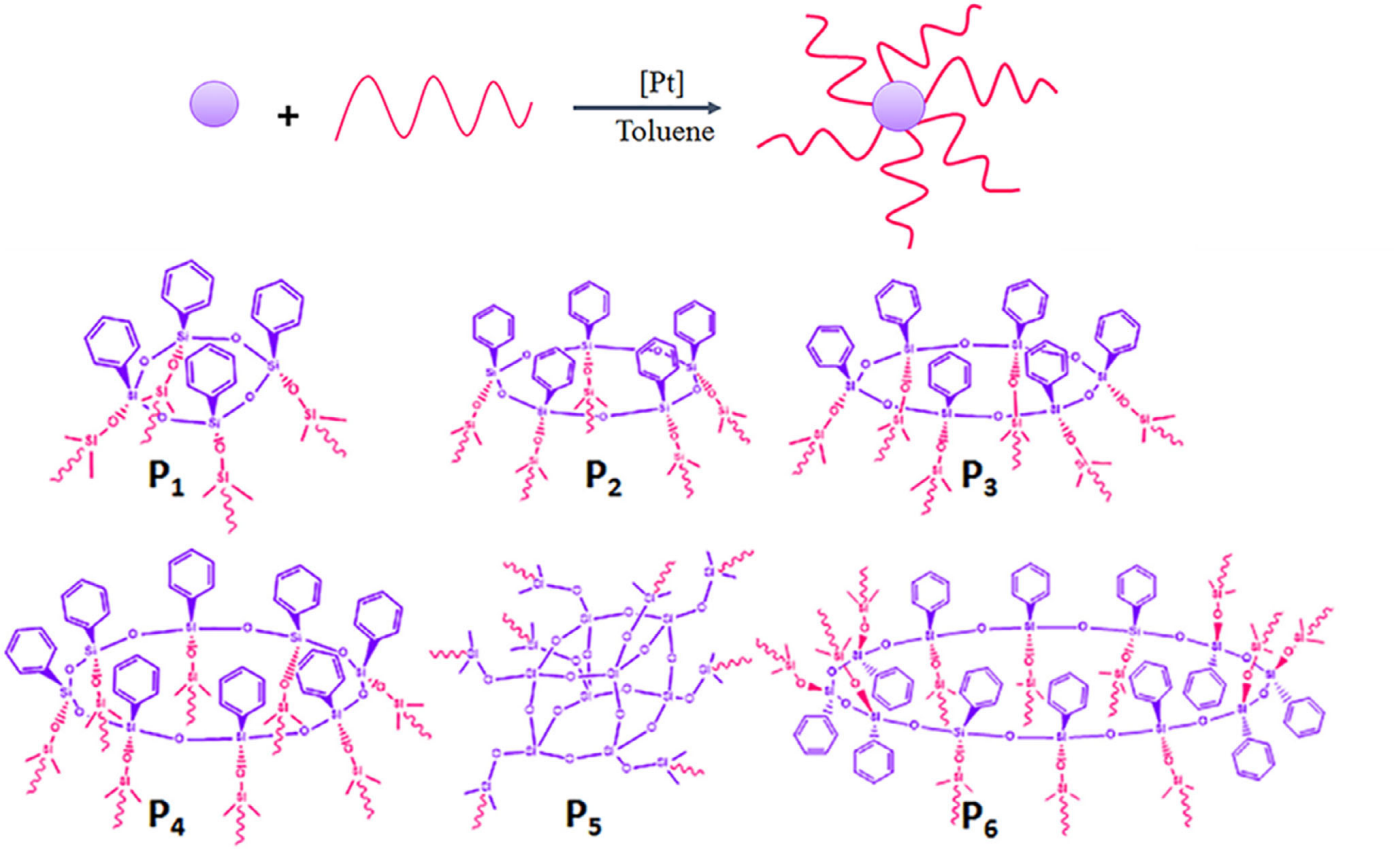
Schematic synthesis pathway toward silsesquioxan‐based star‐branched PDMS and their general architecture depending on the core structure and size. P1: n = 4, P2: n = 5, P3: n = 6, P4: n = 8, P5: n = 8, P6: n = 12. Reprinted with permission from [125]. Copyright 2019 Wiley Periodicals, Inc.

Siloxanes offer a range of cyclic derivatives that are well‐suited as core structures for star‐branched macromolecules, comparable to the hexachlorophosphazene trimer. For example, phenylsilsesquioxan cycles of n = 4, 5, 6, 8, 12, as well as cubic octakis (dimethylsiloxy)‐octasilsesquioxane were applied as functional core structures, and vinyl‐end capped PDMS side chains could be grafted on via hydrosilylation reactions [125]. Depending on the core design, the final star‐branched polymers either exhibited a uniform 3D arm distribution, common for star‐branched architectures, or a so‐called *cis*‐structure, in which all side chains are arranged solely on one side of the core (Figure 35). The materials were extensively characterized and showed comparable results to established star‐branched architectures, although the *cis*‐structures exhibited unexpected rheological behavior in the melt.

**FIGURE 36:**
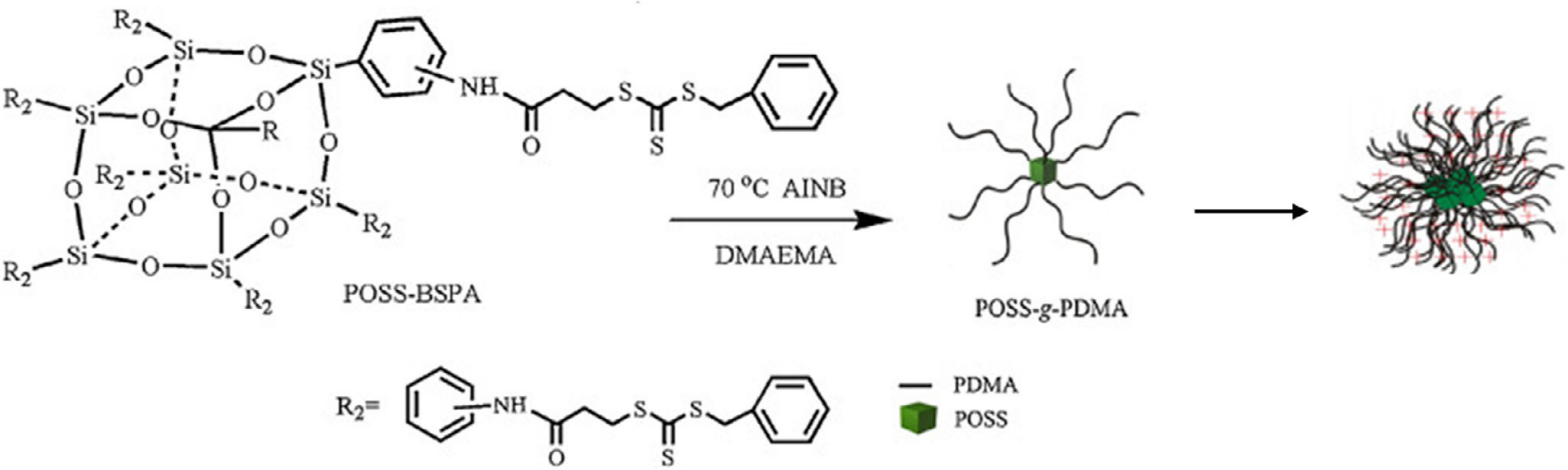
Star‐branched PDMA co‐polymers based on a cubic silsesquioxane and prepared via RAFT polymerization. POSS: polyhedral oligomeric silsesquioxane, BSPA: 3‐benzylsulfanylthiocarbonyl‐sufanylpropionic acid, DMAEMA: dimethylaminoethyl methacrylate. Reprinted from [126], Copyright 2016 Kai Xu, with permission from Elsevier B.V. on behalf of Chinese Chemical Society and Institute of Materia Medica, Chinese Academy of Medical Sciences.

The cubic silsesquioxane was further utilized as a core structure in a grafting‐from approach, benefiting from its rigid, cubic structure, which provides stability and ensures uniform functional group distribution [126]. Octa‐aminophenyl silsesquioxane was functionalized with 3‐benzylsulfanylthiocarbonyl‐propionic acid to produce a reversible addition−fragmentation chain‐transfer (RAFT) agent, which was subsequently used to grow poly(N,N‐dimethylaminoethyl methacrylate) (PDMA) arms in a controlled manner. Driven by the hydrophobic and rigid silsesquioxane cube, the resulting amphiphilic star‐branched polymers self‐assembled into micelles, wherein the cubic silsesquioxane forms the micellar cores and the polymer chains extend as coronas, as shown in Figure 36. The resulting architectures exhibited pH‐responsive behavior, with micellar stability and size strongly depending on solution acidity. Such features highlight their potential for applications as nanocarriers and stimuli‐responsive delivery systems, particularly for controlled drug release in acidic biological environments.

**FIGURE 37:**
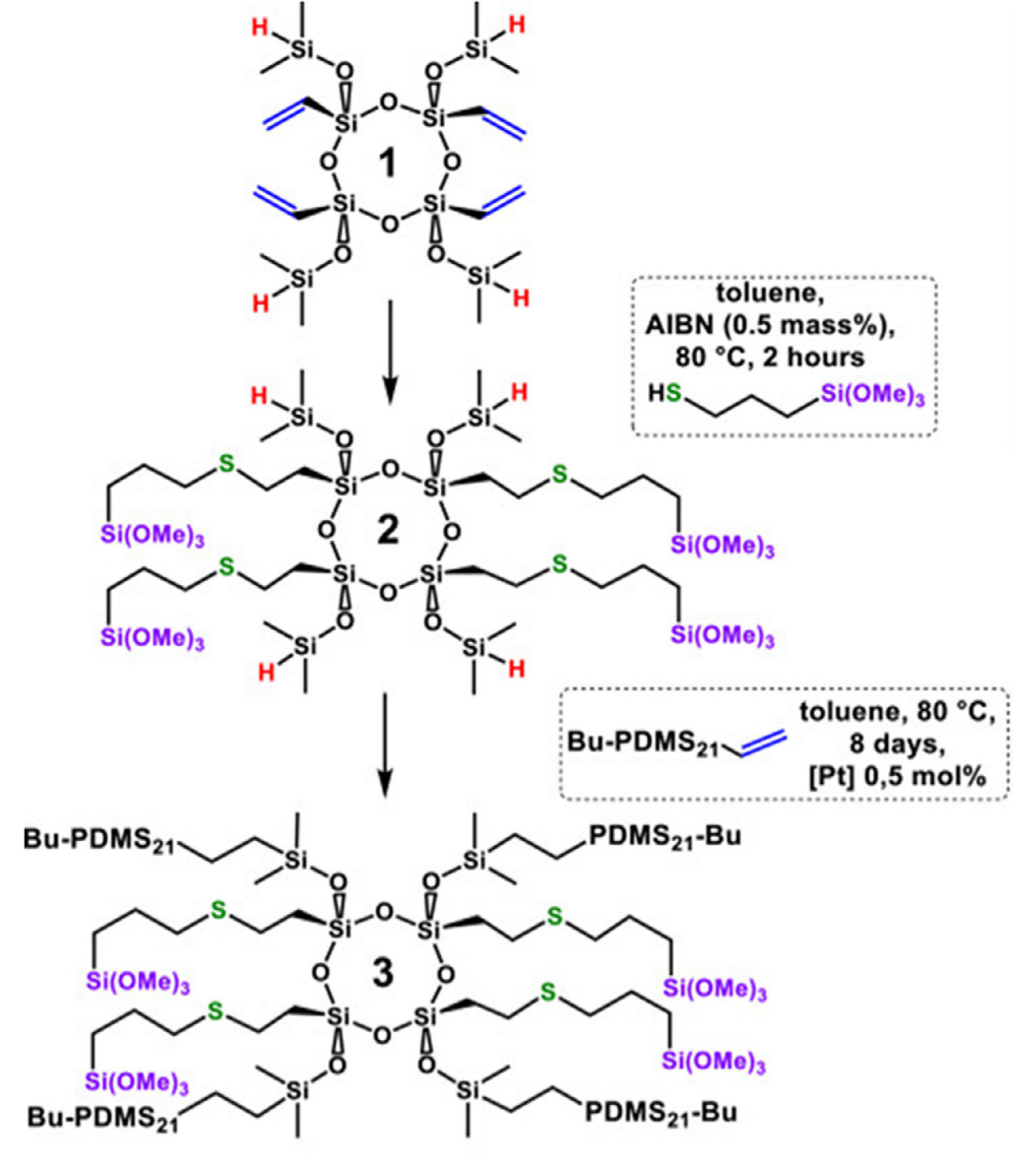
Synthesis of stereoregular Janus star‐branched siloxane macromolecules featuring trimethoxysilane groups and PDMS side chain arms via sequential thiol‐ene functionalization and hydrosilylation reactions, respectively. Reprinted from [127], Copyright 2024, with permission from Elsevier B.V.

Due to the versatility of star‐branched architectures, a distinct approach was employed to synthesize Janus star‐branched siloxane polymers, Figure 37, allowing precise spatial separation of functional groups and enhanced self‐organization [127]. The synthesis involved sequential functionalization of a stereoregular silsesquioxane ring containing Si–H and Si–vinyl groups, introducing a trimethoxysilane derivative and PDMS side chains via a thiol‐ene and a hydrosilylation reaction, respectively, and yielded polymers of high purity and a well‐defined stereoregular structure. In general, the Janus architecture enabled the controlled spatial arrangement of hydrophilic and hydrophobic groups, facilitating advanced self‐organization and showing distinct differences in the properties of such complex compounds when compared to an isomerized star‐branched analogue.

### 4.3 Branched Architectures

**FIGURE 38:**
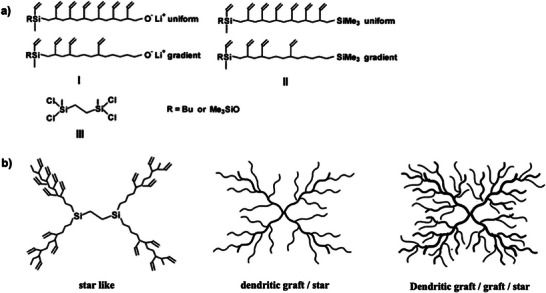
(a) Building blocks and functionalization strategy for the synthesis of branched polysiloxanes. (b) Macromolecular architectures resulting from iterative coupling steps, ranging from star‐like to highly dendritic structures. Adapted with permission from [128]. Copyright 2003 American Chemical Society.

The careful selection of a small subset of building blocks and their well‐considered combination enables the design and iterative build‐up of increasingly complex macromolecular architectures, starting from star‐branched derivatives to dendritic graft/graft/star systems, Figure 38 [128]. The macromolecular building blocks were synthesized via living anionic ring‐opening polymerization, ensuring precise control over the spatial distribution of vinyl groups along the polymer backbone. Branching was subsequently achieved by modification of the vinyl‐functional sites with chlorodimethylsilane and coupling with the polysiloxane chain ends.

**FIGURE 39:**
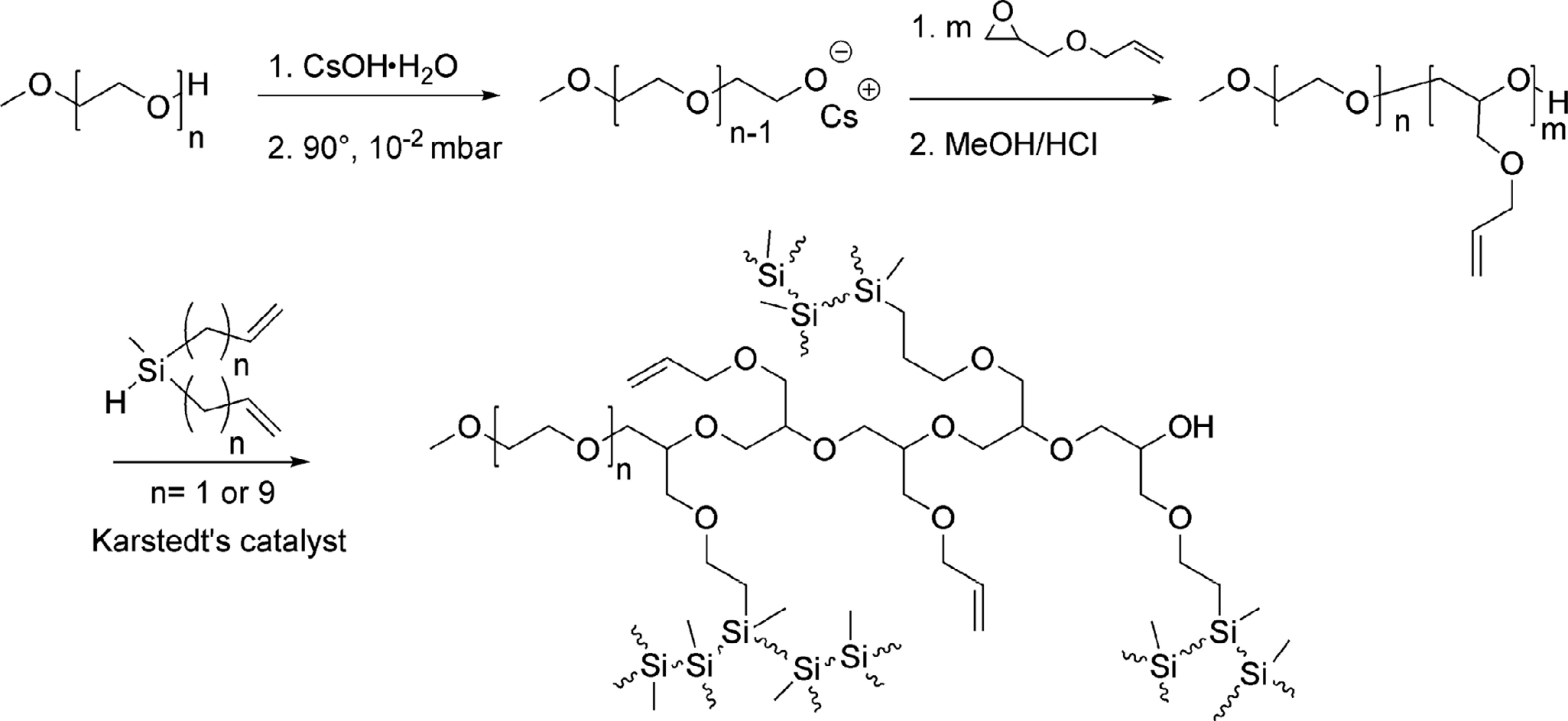
Stepwise synthesis of linear‐hyperbranched block copolymers via anionic ROP of poly(allyl glycidyl ether) from a PEG‐macroinitiator and subsequent hydrosilylation reactions with carbosilane moieties, enabling controlled branching and functionalization. Reprinted (adapted) with permission from [132]. Copyright 2008 American Chemical Society.

Polymerization of trifunctional and tetrafunctional monomers results in the formation of hyperbranched architectures [129, 130]. Although these architectures are not as tightly controlled as described above, they provide a set of interesting properties compared to other designs, and a certain degree of control over their branching can be exercised through Wurtz‐like coupling or sonochemical methods, for example [131]. In one example, amphiphilic linear‐hyperbranched block copolymers, composed of a linear PEG segment and a hyperbranched poly(carbosilane) block, were built on a linear poly(ethylene glycol)‐*b*‐poly(allyl glycidyl ether) block copolymer [132]. Hydrosilylation of an AB_2_‐type carbosilane onto the vinyl groups of the poly(allyl glycidyl ether) block enabled a controlled hyperbranching process, as depicted in Figure 39, resulting in structures exhibiting highly segregated nanophases and anisotropic morphologies.

**FIGURE 40:**
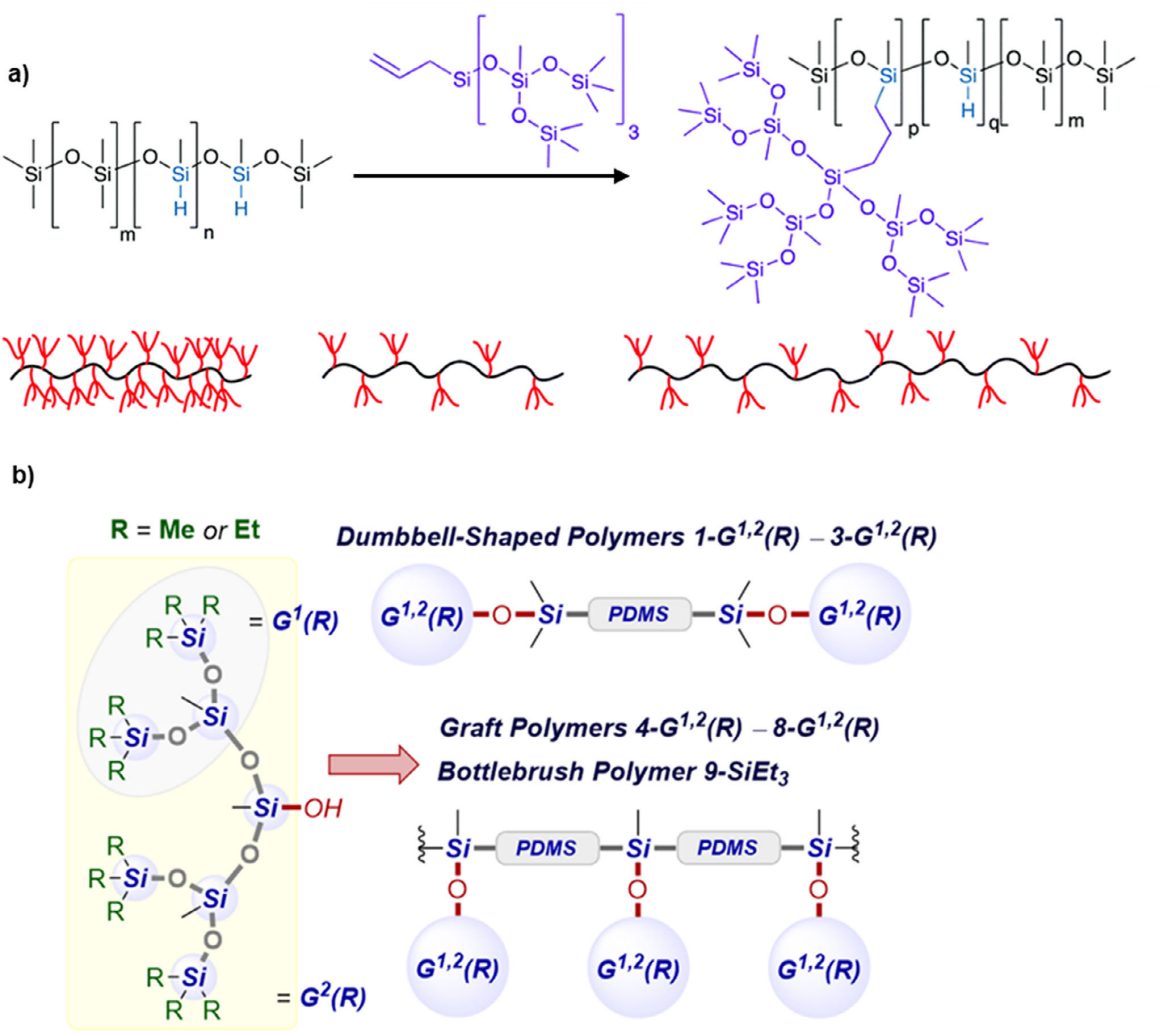
Synthesis of dendron‐decorated PDMS via hydrosilylation reactions and their schematic architectures depending on branching frequency (a) [133], as well as dumbbell‐shaped polymers of all‐siloxane nature (b) [30]. Reproduced from [133] with permission from the Royal Society of Chemistry. Reprinted with permission from [30]. Copyright 2020 Wiley‐VCH GmbH.

Similarly, linear PDMS chains can be decorated with small, monofunctional silicone dendrons, resulting in distinct architectures [133]. Again, a hydrosilylation reaction of the vinyl‐functionalized silicone dendrons with Si─H repeating units in the PDMS backbone provides precise control over branching density and rheological properties, Figure 40a. Interestingly, higher branching frequency initially led to increased viscosity; however, the onset of a globular conformational transition and less efficient interpolymer interactions resulted in a decrease in viscosity. Another approach to achieve such architectures utilizes the Piers–Rubinsztajn reaction, additionally reporting dumbbell‐shaped structures via this chemistry, Figure 40b [30, 134].

Apart from the above‐mentioned examples, Si‐based components such as 4‐(chlorodimethylsilyl)styrene, 4‐(dichloromethylsilyl)diphenylethylene, (chloromethylphenyl)ethyl]methyldichlorosilane, or dimethylchlorosilane were exploited as functional architectural building blocks in various architectures, such as ω‐branched, α,ω‐branched, pom‐pom, H‐shaped, or Janus‐H‐shaped structures, albeit in overall organic macromolecular systems [1, 28, 29, 132].

### 4.4 Dendrimers

**FIGURE 41:**
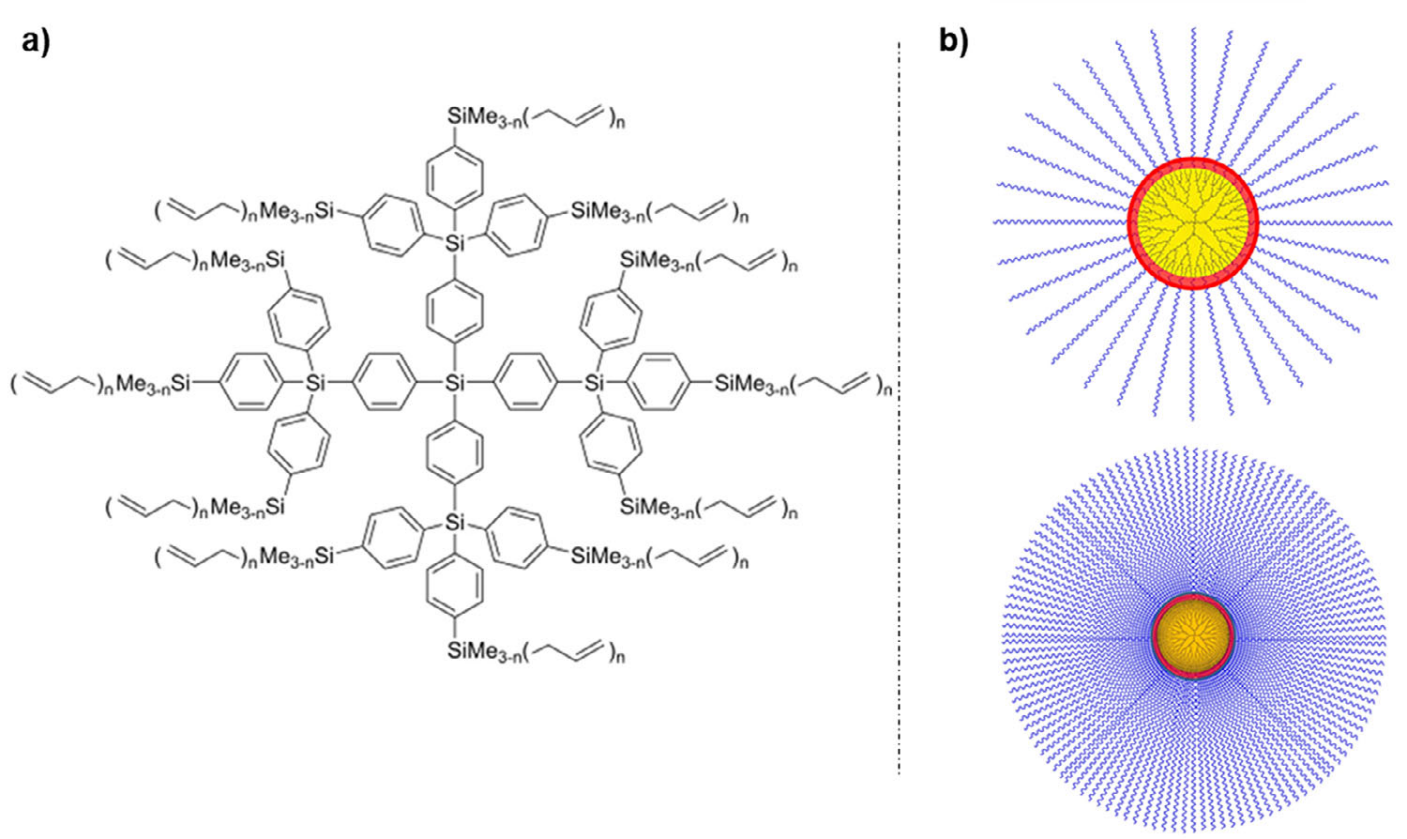
(a) Allyl‐functionalized carbosilane dendrimer with a rigid phenylene core [139]. (b) Schematic representation of carbosilane‐siloxane star‐dendrimer hybrids, illustrating the hierarchical structure with a dendritic core (yellow), an interfacial layer (red), and extended polymer arms (blue) [143]. Reprinted with permission from [139]. Copyright 2013 WILEY‐VCH Verlag GmbH & Co. KGaA, Weinheim. Adapted from [143], licensed under CC BY 4.0. Copyright 2021 by the authors.

The accessibility of various high‐yielding and clean reactions in the field of organosilicon chemistry established these materials as attractive candidates for the preparation of highly controlled, monodisperse dendrimers [135, 136, 137]. While carbosiloxanes predominantly serve as backbones in bottlebrush architectures, carbosilanes represent the dominant dendrimer class due to their high chemical stability, defined 3D structure, and versatile functionalization via robust Si–C bonds [138]. For example, a series of allyl‐end‐grafted carbosilane dendrimers based on 1,4‐phenylene units could be synthesized via a convergent method, Figure 41a [139]. The rigid phenylene spacers provided high structural stability and enabled controlled self‐assembly through C–H···π and π–π interactions, leading to the formation of 3D networks or self‐complementary dimers, as analyzed by NMR spectroscopical methods and crystallography. A divergent approach was used for the preparation of alkyl‐based carbosilane dendrimers, generating each new generation through hydrosilylation reactions of dichloromethylsilane onto an allyl‐moiety and subsequent substitution of the chloride atoms with allylmagnesium chloride [140]. Functionalization of the outer shell further provided surface‐modified dendrimers, including simple butyl chains, a siloxane layer, or lithiation of the allyl groups [141, 142, 143]. The alkyl and siloxane functionalized derivatives exhibited interesting rheological behavior in dependence on their number of generations, drawing attention as new materials. In the case of the polylithium derivative, subsequent ROP of PDMS could be achieved, resulting in star‐branched polymers based on a dendritic core structure, bearing an extensive number of side chains, Figure 41b.

**FIGURE 42:**
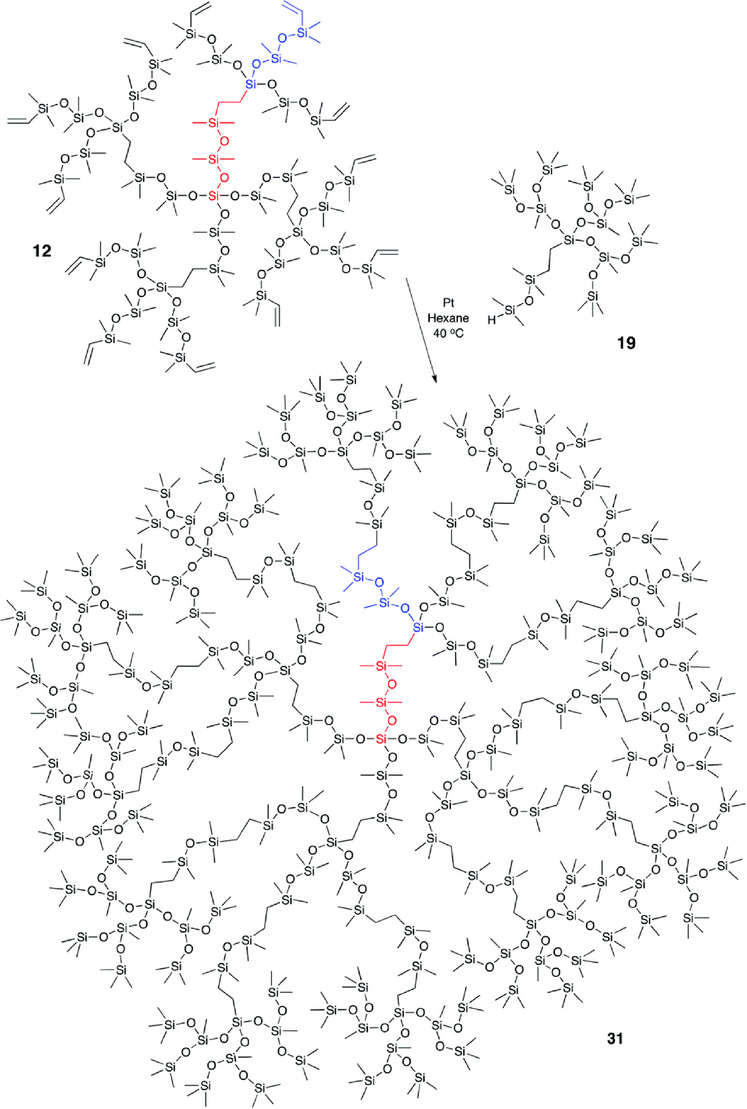
Preparation of carbosiloxane dendrimers through a combination of divergently and convergently prepared dendrons. Reproduced from [144] with permission from the Royal Society of Chemistry.

A siloxane‐based dendrimer was prepared by a well‐thought‐out combination of hydroslilylation reactions and the Piers–Rubinsztajn condensation, avoiding acid/base formation and any accompanying loss of structural integrity via silicone equilibration [144]. Both a convergent and divergent approach could be followed, either starting from a hydrosilylation reaction or via the Piers–Rubinsztajn condensation, yielding highly controlled functional dendrons. Although neither route was suitable for the formation of larger dendrimeric structures, this obstacle could be overcome by combining them, capping divergently generated dendrons with convergently prepared derivatives, Figure 42.

**FIGURE 43:**
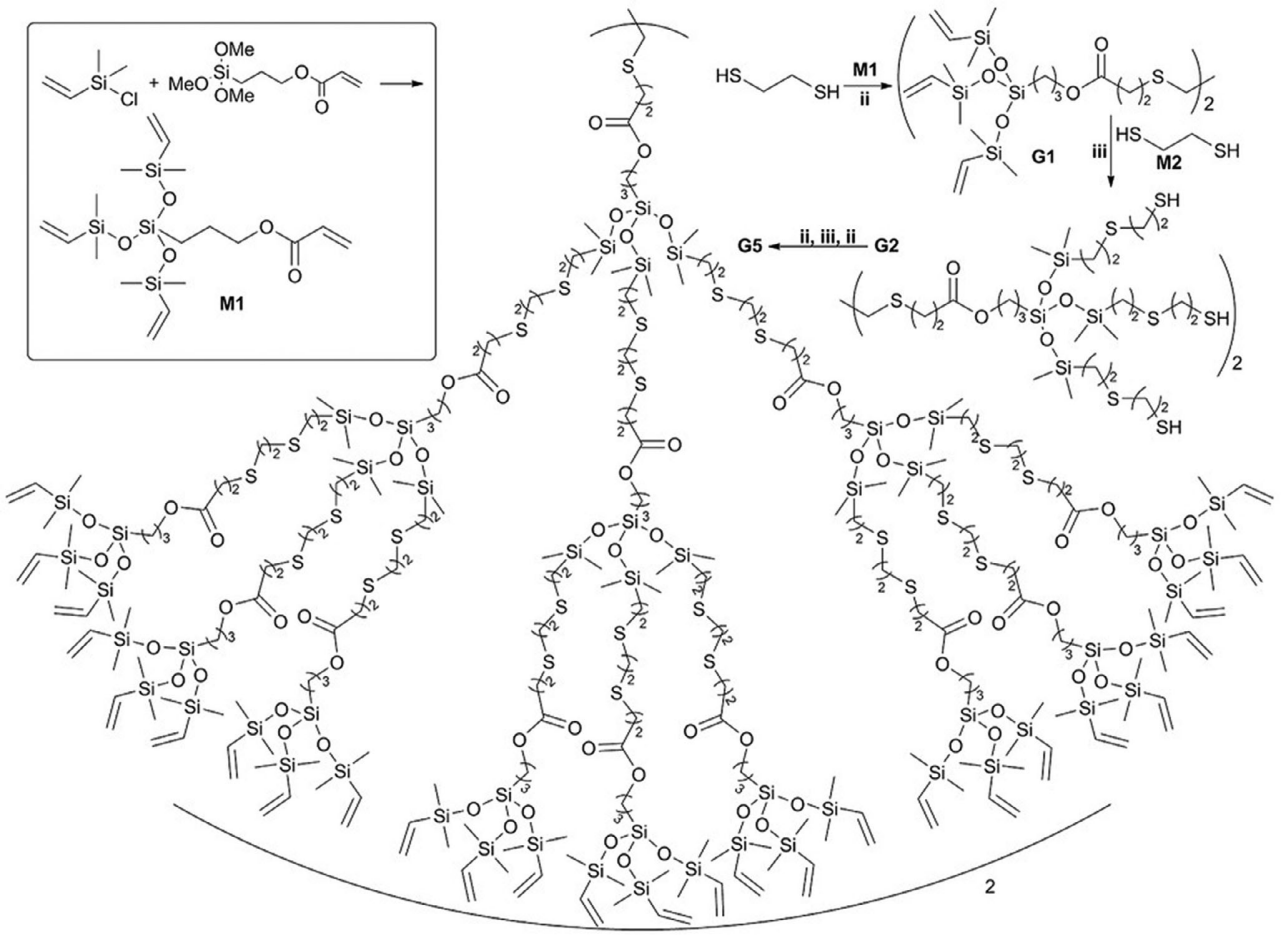
Synthesis of carbosiloxane dendrimers using sequential thiol‐Michael additions and free radical‐mediated thiol–ene addition reactions. ii: **M1**, DBU/THF, room temperature; iii: **M2**, DMPA/THF, room temperature, hυ (365 nm). Reprinted with permission from [145]. Copyright 2015 WILEY‐VCH Verlag GmbH & Co. KGaA, Weinheim.

In a highly efficient, quick, and facile approach, an orthogonal “Click”‐chemistry strategy was employed for the construction of dendrimers up to the fifth generation in less than 24 h under mild conditions [145]. The method takes advantage of the intrinsic features of thiol‐ene chemistry, in detail, the difference in reactivity of thiol‐Michael addition on conjugated C─C double bonds and free‐radical‐mediated thiol–ene addition on remaining electron‐rich C─C double bonds. To this end, a suitable monomer, **M1** in Figure 43, was designed, bearing one conjugated and three unconjugated C─C double bonds, linked by siloxane bonds. The dendrimers were then built up through iterative thiol‐ene reactions, namely, organic base‐catalyzed thiol‐Michael addition and photoinitiated radical‐mediated thiol‐ene reactions, summarized in Figure 43.

Finally, as described already above for star‐branched macromolecular architectures, silsesquioxane derivatives can be utilized as multifunctional core structures for Si‐based dendrimers. Double‐decker silsesquioxane cores have been used for the synthesis of low‐generation dendritic systems (G1, G1.5) via hydrolytic condensation and hydrosilylation reactions, introducing reactive groups for further modifications. This method offers a unique branching topology for highly branched inorganic polymers and expands the toolbox for designing hierarchically structured macromolecular architectures based on silicon [146].

## 5 Architectures of Other Main Group Elements

Apart from phosphorus and silicon, there are few examples of main group elements as integral parts in inorganic polymers for higher macromolecular architectures. Often, such elements are incorporated into the macromolecular design to provide a specific property aside from the architectural characteristic, such as dynamic bonding in the case of boron. Nevertheless, several examples of main group elements beyond phosphorus and silicone will be discussed in the following section.

### 5.1 Sulfur

**FIGURE 44:**
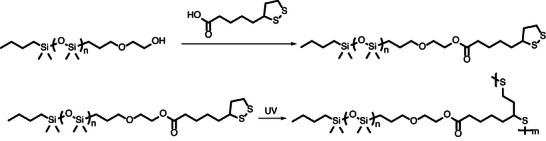
Functionalization and UV‐induced polymerization of α‐lipoic acid modified polysiloxanes [150].

Sulfur generally plays an important role in the field of macromolecular architecture; however, mostly due to its utilization in thiol‐ene addition reactions and vinyl chemistry [147, 148]. Nevertheless, innovative approaches incorporating sulfur in alternative ways are reported in the literature. For example, hyperbranched networks based on polydisulfides were prepared through simple mixing of a dithiol with tris(2‐(pyridin‐2‐yldisulfanyl)ethyl) benzene‐1,3,5‐tricarboxylate under acid catalysis [149]. The thiol‐disulfide exchange directly results in a hyperbranched system, additionally allowing for simple post‐polymerization functionalization via the same thiol‐disulfide exchange on unreacted pyridine‐disulfide motifs. Similarly, the dynamic character of disulfide bonds was used in the formation of bottlebrush elastomers, incorporating α‐lipoic acid as the key building block [150]. Functionalization of a PDMS chain via esterification provides the macromonomer, which can undergo a grafting‐through reaction by simple initiation through UV irradiation in a single‐step process, Figure 44. Incorporation of di‐functionalized chains further enables the direct formation of bottlebrush elastomers, exhibiting exceptionally low shear moduli (20–200 kPa) and allowing light‐induced self‐healing, reprocessing, and targeted chemical degradability due to the reversible nature of disulfide bonds.

### 5.2 Selenium

**FIGURE 45:**
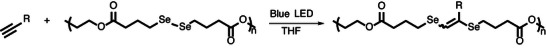
Exemplary reaction equation for the photoaddition of a terminal alkyn into a polydiselenide, allowing the formation of branched macromolecular architectures [152].

Parallel to sulfur, selenium is hardly incorporated in macromolecular architectures as an integral structural building block. One example reports the preparation of a hyperbranched polyselenide through substitution reactions of sodium hydroselenide onto 1,3,5‐tris‐bromomethyl‐2,4,6‐trimethyl‐benzene [151]. Although the prepared material provided promising properties due to the incorporation of Se, containing multiple catalytic sites and hence acting as a glutathione peroxidase mimic, the hyperbranched architectural design was governed by the trivalent aromatic unit. In contrast, a diselenide‐yne system for multifunctional hyperbranched polymers was reported, specifically rooted in the reactivity of diselenides and the formation of seleno radicals under blue light irradiation [152]. Diselenide monomers bearing two alkyne functionalities were designed, acting as AB_2_‐type monomers upon homolytic cleavage of the Se─Se bond, and were directly polymerized upon irradiation with blue light. In an interesting deviation from a conventional grafting‐to approach for the formation of bottlebrush polymers, linear diselenide polymers were reacted with alkyne‐terminated side chains, resulting in a “splicing‐in” of the triple bond into the linear backbone structure, Figure 45. A variety of backbone chemistry as well as side chains could be utilized, further enabling the incorporation of ATRP‐initiator moieties for subsequent “grafting‐from” steps.

### 5.3 Tin

Higher macromolecular architectures of polystannanes remain rare and poorly explored due to their sensitivity to light and susceptibility to degradation [153]. They are primarily synthesized through catalytic dehydropolymerization, allowing for highly controlled linear materials [154], as well as highly branched, high molecular weight polystannanes [155], based on the catalytic system and synthetic approach. On the other hand, organostannic compounds have been developed as building blocks for tin‐centered dendrimers. In particular, bis(triphenylstannyl)‐substituted alkanes have been explored as precursors for organotin dendrimers, where the α,ω‐bis(tri‐(w‐triphenylstannyl)butylstannyl) structure serves as a multifunctional core. These pre‐dendritic architectures are often described as dumbbell‐shaped due to their symmetrical tin‐rich termini and provide potential branching points for further polymerization [156].

### 5.4 Metallocene‐Based Architectures

**FIGURE 46:**
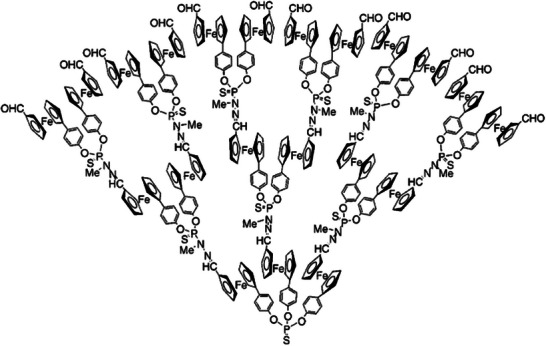
Exemplary structure of a phosphorus‐containing dendrimer incorporating ferrocenyl moieties within the branches. Adapted with permission from [157]. Copyright 2000 American Chemical Society.

Only a limited number of studies explored the direct incorporation of metallocenes into polymer backbones, particularly within highly branched or architecturally complex macromolecular systems. Although some ferrocene‐containing dendrimers are known, research has predominantly focused on the functionalization with ferrocenyl units exclusively onto the surface. Nevertheless, phosphorus‐containing dendrimers with ferrocenyl moieties incorporated directly into the dendritic structure have also been reported, Figure 46 [157]. Such dendrimers were synthesized analogues to the procedure of phosphorhydrazone‐dendrimers, as already described above, except that an asymmetric 1,1′‐disubstituted ferrocene substituted the 4‐hydroxy‐benzaldehyde. Symmetric 1,1′‐ferrocenedicarboxaldehyde could be incorporated as a core structure as well. In general, control over the different ferrocene localizations provides varying oxidative behavior of the structure, making them interesting materials for catalysts or chemical sensors [157, 158].

**FIGURE 47:**
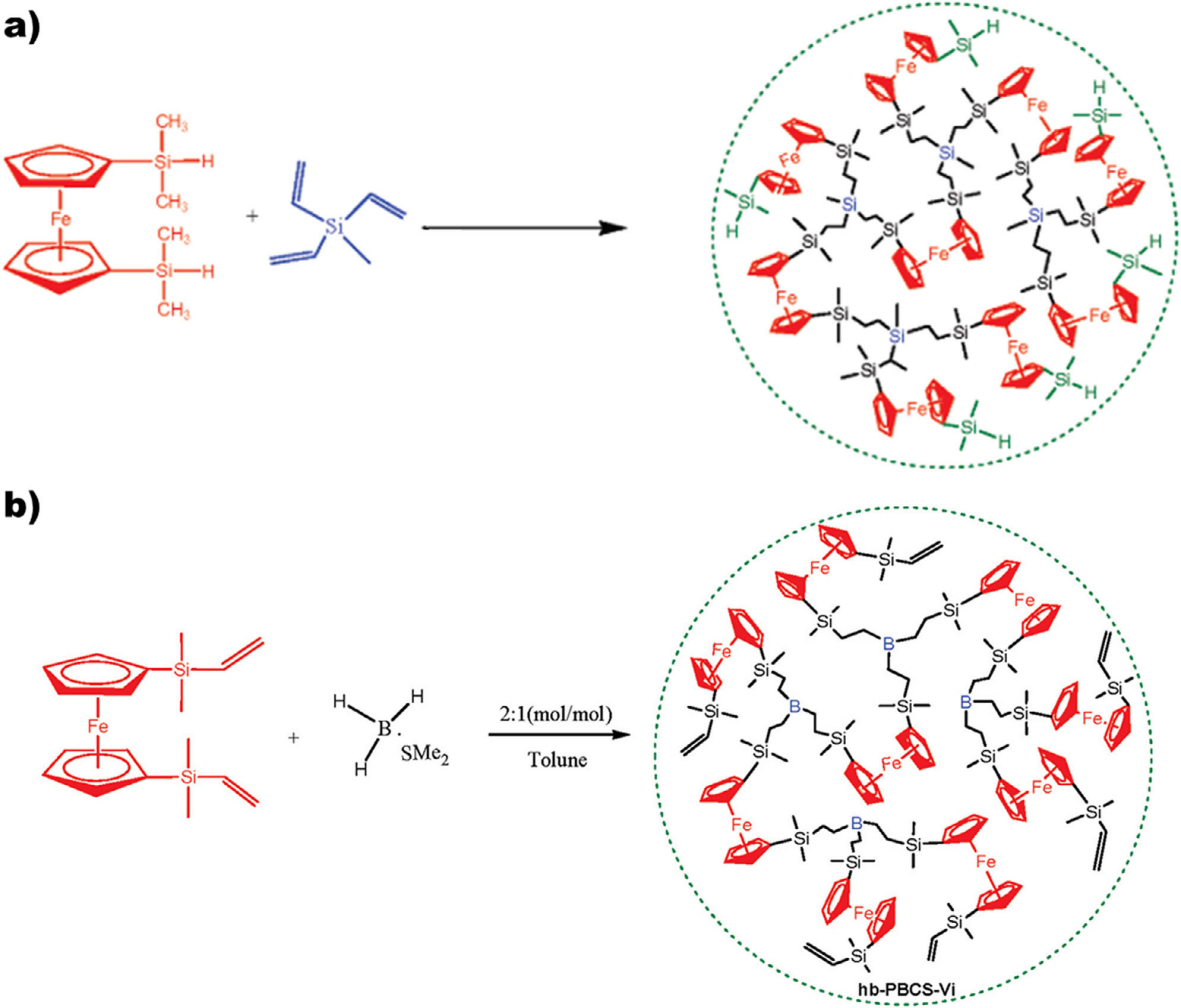
Synthesis of hyperbranched ferrocene‐containing polycarbosilanes (a) and poly(boro)carbosilanes (b). Reprinted (adapted) with permission from [160]. Copyright 2011 American Chemical Society.

Hyperbranched ferrocene‐containing polymers have been reported in combination with silanes, for example, using an “A_2_ + B_3_” approach. In one method, dilithio‐ferrocene was reacted with trichlorisilane in a one‐pot approach, yielding hyperbranched poly(ferrocenylenesilyne). Different substituents on the silicon atom could render the solubility and Tg of the polymer, while the ferrocene introduces magnetic properties, making the material a useful precursor for nanostructured magnetoceramics [159]. Another approach is based on the hydrosilylation reaction of a silyl‐functionalized ferrocene with trivinylmethylsilane, Figure 47a. Adjusting the ratio of the monomers enables post‐polymerization functionalizations, such as the incorporation of boron moieties via hydroboration reactions. Furthermore, borane dimethylsulfide could be incorporated into the polymer backbone, substituting trivinylmethylsilane, Figure 47, adjusting magnetic properties and high‐temperature resistance [160].

Finally, metallocenes incorporating Fe(II), Ru(II), and Zn(II) in their center were also reported as cores for star‐branched polymers. Coordination to functionalized bipyridinyl ligands enables both convergent and divergent routes toward the formation of star‐branched polymers and the use of various polymerization methods for the controlled preparation of the side chains [7, 161].

## 6 Conclusion and Outlook

Inorganic polymers offer access to structural motifs often unattainable through traditional organic polymer chemistry, thanks to their unique elemental compositions and diverse reactivity. As demonstrated throughout this review, a careful selection of elements and functionalities not only provides a vast construction kit for architectural design but also enables fine‐tuning of key material properties such as self‐assembly, responsivity, degradability, and biocompatibility. Phosphorus‐based systems, such as PPz and PPE, offer the possibility of high substitution densities, unmatched side group variety, and biodegradable backbones, particularly relevant to biological applications. In contrast, siloxane‐ and carbosilane‐based materials provide excellent thermal and mechanical stability, well‐suited for designing elastomeric systems. The strategic incorporation of less conventional elements, such as sulfur, selenium, tin, or metallocenes, further expands the design landscape, opening new avenues for the development of functional materials.

A particularly promising strategy involves hybrid materials that combine distinct polymer classes, such as PPz with PDMS, which have proven effective in creating unique, elastic, functionalizable architectures. Nevertheless, the field of inorganic macromolecules remains fragmented, with most advances limited to a handful of well‐established polymer classes. Many other potentially valuable systems remain largely unexplored, and there is a lack of systematic studies that link structural parameters, such as polymer architecture, elemental composition, or side‐chain chemistry, to material performance. In addition, the scalability of complex multi‐step syntheses remains a recognized bottleneck, and the development of more efficient, modular methods will be critical for future translation into commercial products. To accelerate future progress, new synthetic methodologies must be developed to enable the incorporation of a broader range of inorganic elements. Equally important is the development of structure–property relations that allow predictive design. Ultimately, the goal should be to establish generalizable design principles that move the field beyond empirical approaches and support the modular, rational construction of inorganic macromolecules with targeted functionalities. In conclusion, the studies highlighted throughout this review demonstrate that inorganic polymers hold immense, yet underutilized, potential for architectural control in polymer chemistry. Owing to their unique structural diversity and functional potential, they are ideally positioned to become a foundational platform for designing the next generation of advanced materials.

## Conflicts of Interest

The authors declare no conflicts of interest.
